# Deciphering Macroalgal Microbiomes: Metagenomic Insights, Ecological Roles and Bioprospecting Potential

**DOI:** 10.3390/microorganisms14102127

**Published:** 2026-09-22

**Authors:** João Moreira, Raul Bettencourt

**Affiliations:** 1 OKEANOS-Institute of Marine Sciences, University of the Azores, 9901-862 Horta, Portugal; joao.cs.moreira@uac.pt; 2 Faculty of Science and Technology, University of the Azores, 9900-862 Horta, Portugal

**Keywords:** macroalgae, microbiome, holobiont, metagenomics, taxon attribution, CAZymes, polysaccharide utilization loci, biosynthetic gene clusters, bioprospecting, *Cobetia*

## Abstract

Macroalgae host complex microbial communities that influence surface colonization, development, nutrient transformation, carbohydrate turnover, environmental responses, and the production of potentially valuable metabolites. Advances in shotgun metagenomics and genome-resolved analysis have greatly expanded access to this functional diversity, but they have also increased the risk of conflating genomic potential with biological activity or host benefit. This narrative review evaluates the bacterial component of macroalgal microbiomes—the component for which mechanistic and experimentally validated evidence is most extensive—through a framework that separates what has been proven from what is merely predicted, distinguishing functional prediction from expression, biochemical activity, metabolite exchange, host response, and experimental causality. Particular attention is given to bacteria-dependent morphogenesis, metabolic complementarity, environmental acclimation, carbohydrate-active enzymes and polysaccharide-utilization loci, biosynthetic gene clusters, and the translational potential of seaweed-associated microorganisms. Comparative perspectives from microalgal and cyanobacterial systems are used to identify principles that generalize across algal microbiomes while emphasizing the distinctive spatial and chemical complexity of macroalgal surfaces. Invasive and bloom-forming macroalgae provide an additional ecological context in which microbial functional redundancy, recruitment flexibility, and dominant populations may shape whole-community profiles. An illustrative, hypothesis-generating taxon-attribution case study, based on single metagenomic datasets from *Rugulopteryx okamurae* and *Sargassum* sp. holobionts in the Azores, which confounds host identity with sampling location, time, and thallus condition, shows that the disproportionate genomic contribution of the genus *Cobetia* measurably alters the interpretation of a specific subset of community-level KEGG pathway contrasts, while the majority of pathway-level and taxon-specific signals discussed retain their direction after this taxon’s exclusion and residual-community renormalization. Together, these analyses argue that macroalgal microbiome research should move beyond inventories of predicted functions toward explicit taxonomic attribution, experimental validation, and ecological context. Such integration will be essential for establishing which microbial functions are ecologically consequential and which can be translated reproducibly into biotechnology.

## 1. Introduction

Macroalgae harbor complex microbial communities whose members can influence host development, nutrient transformation, surface colonization, stress responses, and the turnover of chemically distinctive marine polysaccharides [1,2,3,4]. Bacteria are by far the most intensively studied component of these communities, and consequently provide most of the mechanistic and experimentally validated evidence available [1,5]; this review accordingly focuses on the bacterial component of macroalgal microbiomes, referring to archaea, fungi, viruses, and microbial eukaryotes only where evidence specific to those groups is discussed. Advances in cultivation-independent sequencing have greatly expanded access to these microbiomes, moving the field from predominantly taxonomic descriptions toward genome-resolved reconstruction of microbial functional potential [4,6].

This transition has also created an interpretive challenge. The detection or relative abundance of a microbial gene or pathway does not establish its expression, biochemical activity, transfer of metabolites to the host, or contribution to host fitness, yet these levels of evidence can be conflated in discussions of macroalgal holobiont function and biotechnology [6,7]. Moreover, whole-community functional profiles can obscure how metabolic capacities are distributed among microbial populations: an apparent holobiont-level difference may reflect a broadly shared community property, a specialized functional guild, or the disproportionate genomic contribution of a single dominant taxon [6,8].

This narrative review examines macroalgal microbiomes through this framework for separating what has been demonstrated from what remains predicted, progressing from experimentally supported host–microbe interactions and metabolic exchange to genome-resolved functional ecology, glycan-degrading systems, natural-product discovery, and microbiome-associated patterns in invasive and bloom-forming macroalgae. This narrative review has two linked purposes: to assess what the current literature establishes about macroalgal microbiome function, and to demonstrate, through an illustrative, hypothesis-generating taxon-attribution case study in Section 9, based on a single metagenomic dataset per host, why taxonomic resolution is required for interpreting community-level metagenomic signals.

Particular emphasis is placed on distinguishing genomic potential from demonstrated function and on linking community-level pathway signals to the microbial populations that encode them. A taxon-attribution case study comparing the metagenomes of *Rugulopteryx okamurae* and *Sargassum* sp. holobionts in the Azores shows that dominance by a single bacterial genus measurably alters the interpretation of a specific subset of whole-community KEGG pathway contrasts, without invalidating the broader comparison. By integrating ecological evidence, metagenomic resolution, and explicit functional attribution, the review argues that the next stage of macroalgal microbiome research will depend less on expanding inventories of predicted functions than on establishing who performs them, when they are deployed, how they affect the host, and which can be translated reproducibly into biotechnology.

### Literature Search and Evidence Classification

The Literature for this narrative review was identified through targeted searches of PubMed/MEDLINE, Web of Science, and Google Scholar, using combinations of the terms “macroalgal microbiome,” “seaweed microbiota,” “algal holobiont,” and “epiphytic bacteria,” together with terms specific to the functional domains covered in each section (e.g., “carbohydrate-active enzymes,” “polysaccharide utilization locus,” “biosynthetic gene cluster,” “metagenome-assembled genome,” “quorum sensing,” and “morphogenesis”). Searches were not restricted to a fixed start date and included both foundational studies establishing key host–microbe interactions and the most recent genome-resolved, multi-omics, and methodological studies available at the time of writing. Reference lists of retrieved articles and existing reviews were also examined for additional relevant studies, and methodological or technical papers not specific to macroalgae, including general metagenomic annotation tools and KEGG pathway databases, were included where necessary to support the analytical framework used in Section 9.

This review does not follow a systematic or scoping-review protocol: no fixed database combination, deduplication procedure, independent dual-reviewer screening, or formal quality-appraisal tool was applied. This is consistent with its stated purpose as a narrative review organized around a conceptual evidentiary framework rather than an exhaustive or protocol-driven synthesis of the literature.

Throughout the review, and particularly in the summary tables, evidence supporting a given microbiome function is classified into one of six tiers according to the evidentiary framework defined here and summarized in Figure 1.

1. Genomic support—a gene, pathway, or biosynthetic gene cluster has been detected via sequencing (amplicon, shotgun metagenomic, or isolate genome sequencing), with no additional experimental validation of expression or activity.
2. Expression evidence—transcriptomic or proteomic data indicate that the gene or pathway is actively expressed, in situ or in culture, beyond its mere genomic presence.
3. Strong biochemical basis—enzymatic activity, metabolite production, or another biochemical function has been directly demonstrated, typically via in vitro assay, heterologous expression, or cultivation of the relevant organism.
4. Metabolite transfer evidence—a specific metabolite has been shown to move between the microbial partner and the host or another community member, for example through stable-isotope tracing or co-culture metabolite quantification.
5. Host response evidence—a measurable phenotypic, physiological, or developmental change in the host has been documented in association with the microbial function in question, for example through comparison of axenic and microbially associated hosts.
6. Experimental/causal evidence—manipulative experiments, such as knockouts, gnotobiotic reconstruction, synthetic community assembly, or equivalent approaches, establish a causal role for the microbial function in the outcome described.

These tiers are not mutually exclusive or strictly cumulative in the literature—a function may have strong biochemical evidence without an accompanying host-response study, for example—but assigning each cited finding to its highest supported tier allows the summary tables throughout this review to distinguish genomic inventories from functionally or causally validated claims.

## 2. Macroalgal Host–Microbiome Systems: From Microbial Diversity to Demonstrated Function

### 2.1. The Macroalgal Holobiont: Host Selection, Environmental Filtering, and Functional Association

Marine macroalgae support diverse microbial communities on and within their tissues, including bacteria, archaea, fungi, protists, and viruses. Among these components, bacteria remain by far the most intensively investigated, and consequently most mechanistic understanding of macroalgal–microbiome interactions derives from bacterial systems. Throughout this review, the term macroalgal microbiome therefore refers primarily to bacterial communities unless other microbial groups are explicitly considered [1,5].

The recognition that these microorganisms can contribute to host-associated processes has encouraged the treatment of macroalgae and their associated microbiota as holobionts rather than as ecologically independent entities [1,9]. The holobiont concept is useful as an ecological framework, but it should not be interpreted as implying that all microbial associates are stable, beneficial, or functionally integrated with the host. Macroalgal surfaces are continuously exposed to microbial immigration from seawater and therefore contain persistent symbionts, transient colonizers, opportunists, and, under some circumstances, pathogens [1,2,5].

A consistent observation across several macroalgal systems is that surface-associated bacterial assemblages differ from those in surrounding seawater, indicating that macroalgal hosts and the physicochemical conditions at their surfaces exert selective pressure on microbial recruitment and persistence [1,2,10]. Host identity, tissue properties, developmental stage, geography, season, and environmental conditions can all influence community structure [2,5,10]. Consequently, reports of recurrent bacterial lineages associated with seaweeds should not be interpreted as evidence for a universal taxonomic “core microbiome.” A more reasonable emerging picture is one in which taxonomic composition may vary substantially among hosts and environments while certain ecological functions recur through different microbial lineages [2,4,11].

This distinction between taxonomic conservation and functional conservation is central to interpreting macroalgal microbiomes. Shotgun metagenomic studies, including genome-resolved analyses of the kelp *Nereocystis luetkeana*, have identified microbial populations with genetic capacities for dissolved-organic-matter transport, vitamin biosynthesis, nitrogen transformations, motility, and polysaccharide utilization [4]. Such observations demonstrate functional potential encoded within the microbiome, but they do not, by themselves, establish that these pathways are expressed in situ or that their products are transferred to and benefit the host. Distinguishing genomic potential from experimentally demonstrated interaction is therefore essential throughout the field.

### 2.2. Bacteria-Dependent Morphogenesis: One of the Strongest Causal Models in Macroalgal Microbiome Research

Among the proposed functions of macroalgal microbiomes, bacterial regulation of development in *Ulva* provides some of the clearest experimental evidence for a causal host–microbe interaction. Axenic cultures of several *Ulva* species develop abnormal morphologies, whereas reintroduction of particular bacterial isolates can restore aspects of normal thallus development [12,13]. These experiments established that normal morphogenesis in this model system depends not simply on bacterial presence but on chemically mediated interactions with specific bacterial partners.

A particularly influential reductionist model involves *Ulva mutabilis* and bacterial representatives related to *Roseovarius* and *Maribacter*. The two bacterial partners exert complementary effects on algal development: one primarily stimulates cell proliferation, whereas the other promotes normal cell differentiation, rhizoid formation, and cell-wall development [12,13]. This division of function provides unusually direct evidence that bacterial metabolites can act as developmental signals across the prokaryote–eukaryote boundary.

One of the best-characterized bacterial morphogens is thallusin, originally isolated from a marine bacterium associated with the green alga *Monostroma* and subsequently implicated in *Ulva* morphogenesis [14,15,16]. Thallusin is active at very low concentrations and can induce developmental responses associated with normal thallus differentiation and rhizoid formation [14,15,16]. Its identification provides a rare example in which a chemically defined microbial metabolite has been linked directly to macroalgal developmental biology.

The complementary morphogenetic activity associated with *Roseovarius* has often been described as cytokinin-like because of its effects on cell division, whereas the developmental response promoted by *Maribacter* has been compared with auxin-like activity. These functional analogies should not be taken to mean that the relevant bacterial signals are necessarily conventional plant cytokinins or auxins. The chemical identity of all components involved in this interaction has not been resolved to the same degree as that of thallusin [12,13].

The *Ulva* system is therefore important not because it proves that bacteria universally control development in macroalgae, but because it demonstrates that such control is possible and can be experimentally dissected. Equivalent bacterial dependence has not been established with comparable mechanistic resolution across brown and red macroalgae. Extrapolation beyond *Ulva* should consequently be made cautiously [5,12,13].

### 2.3. Macroalgal Surfaces as Chemically Structured Microbial Habitats

Macroalgal surfaces provide microbial habitats that differ substantially from the surrounding water column. Dissolved organic compounds released by the host, surface-associated polysaccharides, secondary metabolites, oxygen gradients, and microscale physicochemical conditions create selective environments in which microbial colonization is shaped by both resource availability and chemical interaction [1,17].

Experimental work has shown that macroalgal surface metabolites can influence bacterial settlement, favoring some bacterial associates while deterring others. This process has been described as microbial “gardening,” in which the host contributes to the structuring of its epibiotic community through chemically mediated recruitment and exclusion [17]. Such host filtering provides a plausible mechanism by which beneficial or compatible microorganisms may become enriched at the thallus surface without requiring the assumption of obligate symbiosis.

Microbial competition further contributes to community organization. Surface-associated bacteria can produce antimicrobial metabolites, quorum-sensing molecules, extracellular polymers, and other compounds that affect neighboring microorganisms and may influence colonization resistance. In some systems, bacterial isolates from healthy seaweeds inhibit organisms associated with disease, supporting the hypothesis that members of the resident microbiota can contribute to host protection [1,18].

However, the protective-microbiome concept requires careful interpretation. Detection of antimicrobial activity in bacterial isolates does not necessarily demonstrate protection of the host under natural conditions, and shifts in community composition during disease do not by themselves establish whether microbiome disruption is a cause or a consequence of declining host health. Macroalgal disease is likely to emerge from interactions among host condition, environmental stress, microbial community structure, and opportunistic pathogens rather than from the presence or absence of a single universally protective community [18].

This context dependence is particularly important under environmental stress. Temperature anomalies, nutrient enrichment, pollution, and other disturbances can alter both host physiology and microbial community composition, potentially changing relationships that are beneficial or neutral under one set of conditions into antagonistic ones under another [3,18]. Macroalgal–microbial associations are therefore better understood as dynamic ecological relationships distributed along a continuum from mutualism to antagonism, rather than as fixed symbiotic states.

#### Distinguishing Compartments, States, and Timepoints Within the Macroalgal Holobiont

Much of the literature reviewed above treats “the macroalgal microbiome” as a single entity, but several dimensions of variation are increasingly recognized as biologically consequential and are not interchangeable.

Epiphytic versus endophytic communities. The surface-associated (epiphytic) community, typically sampled by swabbing or washing, is by far the most extensively characterized component of the macroalgal holobiont [2,19]. Bacteria residing within algal tissue (endophytic) are comparatively understudied, in part because their recovery requires surface sterilization and tissue disruption rather than surface sampling, and the two compartments can differ substantially in composition even within the same host individual [19]. Conclusions drawn from epiphytic sampling, including much of the evidence summarized in this review, should not be assumed to describe the endophytic compartment.

Healthy microbiome versus pathobiome states. As discussed above, shifts in community composition during disease do not by themselves establish whether microbiome disruption is a cause or a consequence of declining host health. The “pathobiome” concept—a disease-associated microbial community that differs from the community of a healthy host—is analytically distinct from a healthy baseline microbiome, and studies that do not explicitly compare diseased and healthy tissue from the same host and location cannot reliably distinguish the two states [18,20].

Tissue-specific variation. Where it has been examined directly, epiphytic community composition differs measurably among tissue types and even between sexes within a single macroalgal species. In *Sargassum thunbergii*, tip, receptacle, and holdfast tissues each supported distinguishable epiphytic bacterial communities, with additional differences detected between male and female thalli [21]. Findings from a single tissue or a pooled whole-thallus sample should therefore not be generalized to the whole organism without qualification.

Life-cycle stage. Macroalgae with alternating haploid and diploid life-history phases (e.g., gametophyte/sporophyte) can host distinct microbial communities at each stage. In the red alga *Mastocarpus* spp., gametophyte and sporophyte life-history phases supported significantly different surface bacterial communities despite both being recovered from the same intertidal sites [22]. In the kelp *Ecklonia radiata*, gametophyte and parental sporophyte microbiota were distinct from the surrounding water column but similar to each other, consistent with a degree of microbiota transmission across life-history stages [23]. Sampling a single life-history stage, as in the present review’s own Section 9 case study, therefore captures only one point in a potentially variable developmental trajectory.

Seasonal patterns. Epiphytic bacterial community composition is also influenced by seasonal, environmental, and physiological variation in the host [2], meaning that single-timepoint sampling—again including Section 9 case study in this review—cannot distinguish a stable host-associated signature from a seasonally transient one.

Collectively, these distinctions indicate that “the macroalgal microbiome” is better understood as a family of related but distinguishable community states—defined by compartment, health status, tissue, life-cycle stage, and season—rather than a single, stable entity attributable to a host species as a whole.

### 2.4. Functional Interaction Does Not Imply Universal Dependence

The increasing use of the holobiont framework has occasionally encouraged language suggesting that macroalgae universally depend on their microbiomes for normal function. Current evidence does not justify such a generalization. The strength of host–microbe dependence varies among species, biological processes, and environmental contexts [1,5].

At one end of the evidence spectrum, experiments with axenic *Ulva* demonstrate a direct requirement for bacterial signals during normal morphogenesis [12,13]. At the other, many functions proposed from metagenomic datasets—such as vitamin provisioning, nitrogen transformation, sulfur metabolism, or polysaccharide recycling—are inferred from the presence of microbial genes and metabolic pathways rather than from direct demonstration of metabolite exchange with the host [4,11].

This distinction follows the evidentiary hierarchy defined in Figure 1.

Much of contemporary macroalgal microbiome research lies toward the first half of this sequence [5]. Genomics and metagenomics are exceptionally powerful for identifying candidate interactions, but stronger causal inference requires integration with metatranscriptomics, metabolomics, stable-isotope tracing, microbial manipulation, synthetic communities, or other experimental approaches capable of linking microbial function directly to host phenotype.

Recognition of these evidential limits does not diminish the importance of macroalgal microbiomes. Rather, it defines the central challenge now facing the field: moving from descriptions of who is present and what they could potentially do toward determining which organisms perform particular functions, under which environmental conditions, and with what consequences for the host.

This challenge becomes particularly important when microbiomes contain highly abundant individual taxa. Community-level functional profiles can then reflect either broadly distributed metabolic capabilities or the genomic contribution of one dominant population. Discriminating between these alternatives requires taxon-resolved functional analysis [6,8], an issue revisited later in this review in the context of invasive macroalgal holobionts.

### 2.5. Beyond Bacteria: An Important Blind Spot in the Current Holobiont Literature

Although macroalgal holobionts include multiple microbial domains, current mechanistic understanding remains strongly biased toward bacteria. Archaea, fungi, viruses, and other microbial eukaryotes are increasingly detected in association with seaweeds, but their ecological and functional contributions remain much less resolved [5]. Archaeal ammonia oxidizers have been identified on several macroalgal species [24], and genome-resolved studies have recovered archaeal populations with predicted nitrogen- and sulfur-associated metabolic capacities [11]. Viral communities are likewise emerging as a substantial but understudied component of seaweed holobionts, with recent viromic studies revealing both host-associated viral diversity and auxiliary metabolic genes potentially affecting microbial and carbon-processing functions [25].

### 2.6. Scope and Conceptual Framework of This Review

The evidence summarized above establishes three principles that guide the remainder of this narrative review. First, macroalgal-associated microbial communities are structured habitats rather than random subsets of surrounding seawater. Second, experimentally demonstrated microbial effects exist, but mechanistic resolution is highly uneven across hosts and biological processes. Third, genomic detection of functional pathways must be distinguished from their expression, ecological deployment, and physiological consequences.

The following sections therefore examine macroalgal microbiome function at progressively different levels of evidence. We first consider metabolic exchanges and environmental responses inferred or demonstrated at the algal–microbial interface. We then evaluate the genomic approaches used to reconstruct microbial functional potential, including metagenome-assembled genomes, carbohydrate-active enzymes, polysaccharide-utilization loci, and biosynthetic gene clusters. Finally, we ask how these approaches can be translated into biotechnology without conflating sequence prediction with validated activity.

This distinction becomes especially important in invasive and bloom-forming macroalgae, where functional differences between holobionts may arise either from community-wide shifts or from the disproportionate contribution of individual dominant microorganisms. Separating these two sources of functional variation provides the conceptual basis for the taxon-resolved analyses considered later in this review.

## 3. Metabolic Exchange and Environmental Acclimation at the Macroalgal–Microbial Interface

Macroalgal surfaces are chemically and physically structured habitats in which host-derived compounds, microbial metabolism, and environmental gradients interact over small spatial scales. Photosynthetic carbon release, dissolved nutrients, extracellular polymers, secondary metabolites, and local redox conditions collectively influence the microorganisms that colonize the thallus and the functions they are capable of performing. Conversely, associated microorganisms can transform compounds within this boundary layer and thereby modify the chemical environment experienced by the host [1,4,17].

The existence of these metabolic capabilities is increasingly supported by cultivation studies, genome-resolved metagenomics, comparative genomics, and experimental host–microbe systems. What is less consistently established is whether particular metabolites are transferred directly from microorganisms to the host, whether these exchanges improve host performance, and under which environmental conditions they become ecologically important. A gene encoding a metabolic pathway demonstrates biochemical potential; it does not, by itself, demonstrate pathway expression, metabolite exchange, or host benefit [4,6,7]. This distinction is fundamental when interpreting metabolic interactions within macroalgal holobionts.

### 3.1. Host-Derived Carbon and Microbial Resource Acquisition

Macroalgae release a fraction of their photosynthetically fixed carbon into the surrounding water as dissolved organic matter. This material can include carbohydrates, sugar alcohols, organic acids, amino acids, and other low-molecular-weight compounds whose composition varies among algal lineages, tissues, physiological states, and environmental conditions [26,27,28]. These exudates generate locally enriched carbon resources that can support heterotrophic microbial populations at the thallus surface.

Genome-resolved metagenomic studies support this interpretation. In the microbiome of the bull kelp *Nereocystis luetkeana*, bacterial metagenome-assembled genomes contained numerous transport systems potentially involved in the acquisition of dissolved organic substrates, including ATP-binding cassette (ABC) and tripartite ATP-independent periplasmic (TRAP) transporters [4]. Members of the genus *Granulosicoccus*, among the prominent kelp-associated Gammaproteobacteria identified in that study, possessed particularly extensive repertoires of transport-related genes.

These genomic features are consistent with adaptation to an environment in which diverse organic compounds are continuously or intermittently available. They do not, however, reveal precisely which substrates are consumed in situ, whether those compounds originate directly from the algal host, or the extent to which individual microbial populations depend upon them [4].

The carbon relationship should therefore not be reduced to a simple reciprocal transaction in which the host supplies photosynthates in exchange for defined microbial services. Such exchanges may occur, but at the community level the available evidence more securely demonstrates host-associated resource availability and microbial utilization potential. Resolving actual carbon fluxes will require approaches capable of linking microbial identity with substrate utilization, including stable-isotope probing, exometabolomics, spatial chemical measurements, and experimentally reconstructed host–microbe systems.

### 3.2. Vitamin B12 and Nutritional Complementarity

Vitamin B12, or cobalamin, provides an important example of possible metabolic complementarity between algae and associated bacteria. De novo cobalamin biosynthesis is restricted to prokaryotes, whereas many algal species possess B12-dependent metabolic pathways and obtain the vitamin from external sources [29,30]. However, B12 dependence is not universal among algae. Different algal lineages vary in their reliance on cobalamin-dependent metabolism, and some retain alternative B12-independent enzymes. It is therefore more appropriate to ask which macroalgal hosts are physiologically dependent on external cobalamin, which members of their associated microbiota are capable of producing it, and whether biologically significant transfer occurs between them.

Metagenomic evidence indicates that such interactions are plausible. In the *Nereocystis luetkeana* microbiome, bacterial metagenome-assembled genomes (MAGs) belonging to several Proteobacterial lineages encoded genes associated with cobalamin biosynthesis. These findings demonstrate that members of the kelp-associated microbial community possess the genetic capacity to produce B12. They do not independently demonstrate transfer to the algal host [4].

Experimental work in algal–bacterial systems has nevertheless shown that bacterial cobalamin production can satisfy algal B12 requirements, establishing microbial vitamin provisioning as a biologically realistic mechanism [29]. Whether equivalent exchanges operate quantitatively within individual macroalgal holobionts remains host-specific and requires direct experimental confirmation.

Vitamin B12 therefore represents a useful example of the distinction between metabolic complementarity and demonstrated nutrient exchange: bacterial biosynthetic capacity can make provisioning possible, but the ecological importance of that capacity depends on host physiology, pathway activity, metabolite availability, and actual transfer between partners [4,29,30].

### 3.3. Microbial Nitrogen Transformations at the Thallus Surface

Nitrogen availability is an important determinant of macroalgal growth and productivity. At the macroalgal surface, associated microorganisms can potentially transform nitrogen among oxidized, reduced, and organic forms, thereby modifying the chemical environment within the host boundary layer.

Genome-resolved studies have identified genes associated with nitrate and nitrite reduction, urea utilization, ammonium transport, and related nitrogen-transforming pathways within macroalgal-associated microbial communities. In *Nereocystis luetkeana*, for example, several bacterial MAGs contained genes involved in nitrate reduction and urea hydrolysis, including nitrate- and nitrite-reduction components and urease systems [4]. These pathways demonstrate the capacity of associated microorganisms to transform nitrogen compounds within the kelp-associated environment. Such processes could modify the relative availability of nitrate, nitrite, ammonium, urea-derived nitrogen, and other nitrogenous substrates near the thallus. The presence of these pathways does not, however, demonstrate direct provisioning of nitrogen to the host [4].

A conservative interpretation is therefore that microbial metabolism can modify local nitrogen speciation and may consequently influence nutrient availability to both the macroalgal host and other members of the microbiome. The ecological consequences will depend on environmental nutrient concentrations, diffusion within the surface boundary layer, host uptake kinetics, and competition among microorganisms.

The same evidentiary distinction applies to nitrogen fixation. Detection of nitrogenase genes such as *nifH* establishes nitrogen-fixation potential but does not, by itself, demonstrate active fixation. Functional evidence requires measurements of nitrogenase activity, N_2_ incorporation, transcriptional activity under appropriate conditions, or equivalent approaches. Demonstrating transfer of newly fixed nitrogen to the host represents an additional level of evidence beyond establishing microbial nitrogen fixation itself.

Importantly, stronger experimental evidence has recently been obtained in an *Ulva* system. Nitrogen-fixing *Cobetia* isolates carrying *nifH* exhibited measurable nitrogenase activity and, when co-cultured with axenic *Ulva prolifera*, supplied biologically available nitrogen and enhanced algal growth [31]. This provides substantially stronger evidence than gene detection alone because microbial nitrogen fixation, host-associated nitrogen provisioning, and a measurable host response were demonstrated experimentally.

### 3.4. DMSP Links Host Physiology, Microbial Behavior, and Sulfur Metabolism

Dimethylsulfoniopropionate (DMSP) is an important organosulfur compound in marine environments and is produced by numerous algal taxa. Depending on species and physiological context, DMSP has been associated with osmotic regulation, oxidative stress responses, and other aspects of sulfur and stress physiology [32,33]. Once released from algal cells, DMSP can also serve as an important carbon and sulfur substrate for marine bacteria, linking algal physiology with microbial sulfur metabolism [34]. DMSP can additionally act as a chemoattractant for motile marine microorganisms [35], providing a mechanistic basis by which localized DMSP release could influence microscale microbial behavior near algal surfaces. Evidence for such chemoattraction does not, however, by itself establish host-specific microbial recruitment on macroalgal thalli.

Microbial DMSP metabolism proceeds through distinct biochemical routes with different ecological consequences. In the demethylation pathway, DmdA catalyzes the initial transformation of DMSP and channels sulfur toward downstream assimilation pathways. This route does not directly generate dimethyl sulfide [34]. In contrast, DMSP cleavage, mediated by Ddd-family enzymes in bacteria results in the formation of dimethyl sulfide (DMS) [36]. DMSP-cleaving lyase activity also occurs in algae, providing an additional biological route to DMS formation [37].

This biochemical distinction is essential when interpreting metagenomic datasets. Detection of *dmdA* indicates the potential for DMSP demethylation, whereas evidence for biological DMS production requires the presence or activity of cleavage pathways [34,36,37]. DMSP therefore provides an instructive example in which the presence of different pathway components can imply substantially different ecological outcomes.

The ecological significance of DMSP extends beyond sulfur transformation itself. Host-derived DMSP can potentially connect algal physiological state with microbial chemotaxis, substrate utilization, and community composition [32,33,34,35]. Environmental stress may alter DMSP production or release, potentially modifying the resource landscape encountered by microorganisms capable of responding to or metabolizing this compound.

Such interactions should not automatically be interpreted as mutualistic. DMSP can benefit microorganisms as a nutrient source without necessarily producing a reciprocal benefit for the host. The consequences of DMSP-mediated interactions therefore depend on the organisms involved and the environmental context.

### 3.5. Microbiome Contributions to Environmental Acclimation

Environmental conditions strongly influence both macroalgal physiology and microbiome composition. Temperature, salinity, nutrient availability, irradiance, desiccation, and contaminants can modify the physicochemical properties of the thallus surface and consequently affect microbial recruitment and activity [3,18].

The observation that microbiome composition changes during environmental stress does not by itself demonstrate microbiome-mediated host acclimation. Environmental disturbance can simultaneously affect host physiology and microbial community structure, making causal direction difficult to establish. Stronger evidence comes from experimental systems in which the microbiota are manipulated and the resulting host phenotype is measured.

#### 3.5.1. Cold Environments and Maintenance of Developmental Function

Cold-adapted macroalgal systems provide evidence that associated bacteria can retain ecologically relevant functions under low-temperature conditions. Studies of Antarctic *Ulva* and its associated microorganisms have shown that cold-adapted bacterial isolates can maintain algal growth- and morphogenesis-promoting activity at temperatures characteristic of polar environments [38]. These experiments extend the bacteria-dependent morphogenesis model described in Section 2 by demonstrating that particular microbial partners remain capable of supporting algal development under severe thermal constraints.

This evidence differs fundamentally from the simple detection of cold-adaptation genes in associated bacteria. Cold-shock proteins, compatible-solute pathways, changes in membrane composition, and other adaptations explain primarily how microorganisms themselves maintain cellular function at low temperature. Their presence does not establish direct transfer of cryoprotective molecules to the macroalgal host.

The evidence therefore supports the conclusion that cold-adapted bacteria can preserve microbiome-dependent developmental functions under low-temperature conditions, rather than the broader claim that macroalgal microbiomes generally provide direct cryoprotection to their hosts [38].

#### 3.5.2. Salinity Acclimation in Ectocarpus

The brown alga *Ectocarpus subulatus* provides one of the stronger experimental examples of microbiome-dependent environmental acclimation. Dittami et al. demonstrated that disruption of the associated microbiota impaired the ability of *Ectocarpus* cultures to acclimate to reduced salinity, whereas restoration of the microbiota restored acclimatization capacity [39]. Because manipulation of the microbial community altered host performance, this experiment provides evidence for a causal microbial contribution rather than a simple correlation between environmental conditions and microbiome composition.

The precise mechanisms responsible for this effect remain less well determined. Changes in microbial metabolism, chemical signaling, nutrient availability, or host physiological regulation may all contribute, but the available evidence does not yet allow a single mechanism to be assigned confidently [39].

The *Ectocarpus* system therefore illustrates an important distinction in the evidence hierarchy: the contribution of the microbiome to the host phenotype is experimentally supported, while the biochemical basis of that contribution remains incompletely characterized [39].

#### 3.5.3. Metal Exposure and Detoxification

Marine microorganisms possess diverse mechanisms for surviving exposure to metals, including efflux systems, extracellular binding, intracellular sequestration, enzymatic redox transformations, and, for some elements, volatilization. Extracellular polymeric substances within bacterial biofilms can also modify metal adsorption and speciation at biological surfaces [40]. Macroalgal-associated bacteria possessing these capabilities may consequently influence metal concentrations and chemical forms in the immediate thallus environment. Such processes are of ecological interest and may also have biotechnological relevance for bioremediation [40].

However, microbial resistance to metals, microbial transformation or removal of metals, and demonstrated protection of the macroalgal host represent different levels of evidence. Studies showing metal binding, sequestration, or transformation by *Bacillus*, *Pseudomonas*, and other marine bacteria establish microbial capability. They do not necessarily demonstrate that macroalgae depend on these microorganisms for survival in contaminated environments [40]. Similarly, algal–bacterial consortia capable of removing chromium, lead, cadmium, or other metals may have bioremediation potential without necessarily representing evolved host-protective symbioses [40]. 

The most plausible interpretation is therefore that associated microorganisms can modify metal chemistry in the immediate algal environment and may, under some circumstances, reduce host exposure. Direct microbiome-mediated protection of macroalgae against metal toxicity remains less extensively demonstrated [40] (Table 1).

### 3.6. An Evidence Hierarchy for Metabolic Interaction in Macroalgal Holobionts

As defined in Figure 1 and discussed in Section 2.4, proposed metabolic interactions can be evaluated along a continuum from genomic potential through expression and biochemical activity to metabolite transfer, host response, and experimental causality. This framework is particularly useful for distinguishing plausible metabolic complementarity from demonstrated symbiotic function.

The *Ulva* morphogenesis systems and the salinity-acclimation experiments in *Ectocarpus* illustrate cases in which microbial manipulation has established a direct contribution to host phenotype. By contrast, many proposed interactions involving bacterial vitamin synthesis, nitrogen transformations, sulfur metabolism, or other metabolic pathways remain supported primarily by genomic capacity or biochemical plausibility rather than direct evidence of transfer to the host.

This distinction should guide interpretation throughout the remainder of the review: metagenomic data can identify candidate interactions and functional potential, but stronger ecological inference requires evidence that those functions are expressed, biochemically active, and consequential for the host.

### 3.7. From Metabolic Inventories to Interaction Networks

Macroalgal microbiome studies have advanced rapidly from taxonomic inventories toward genome-resolved descriptions of metabolic potential. These approaches have revealed microbial communities capable of processing host-associated carbon compounds, synthesizing vitamins, transforming nitrogen and sulfur compounds, degrading complex polysaccharides [4,11].

The next challenge is not simply to identify additional genes and pathways, but to determine which organisms perform particular functions, when those functions are active, which metabolites move between partners, and whether those exchanges affect host performance.

Achieving that transition will require greater integration of shotgun metagenomics with metatranscriptomics, metaproteomics, metabolomics, stable-isotope tracing, spatially resolved chemical imaging, microbial cultivation, and experimentally reconstructed communities [6,7]. Such approaches can distinguish metabolic potential from ecological activity and identify interactions that are genuinely reciprocal rather than merely co-occurring.

This distinction becomes especially important when microbial communities are strongly uneven in taxonomic composition. A pathway that appears abundant at the whole-community level may represent a broadly distributed functional property, or it may predominantly reflect the genomic contribution of one highly abundant microbial population. Community-level pathway abundance alone cannot distinguish between these possibilities [6,8].

Genome-resolved and taxon-resolved approaches are therefore essential for moving from functional inventories toward mechanistic interpretation [4,6]. The following section examines how metagenomics and metagenome-assembled genomes are beginning to provide that resolution.

Evidence-strength legend: Genomic support—gene/pathway/BGC detected by sequencing only. Expression evidence—transcriptomic/proteomic confirmation of activity. Strong biochemical basis—enzymatic activity or metabolite production demonstrated directly. Metabolite transfer—demonstrated exchange between microbial partner and host/community. Host response—documented host phenotypic/physiological change. Experimental/causal evidence—manipulative experiment establishes causality. See “Literature Search and Evidence Classification” (Introduction) for full tier definitions.

## 4. Resolving Functional Potential in Macroalgal Microbiomes: Metagenomics, MAGs, and Bioprospecting

Culture-independent sequencing has transformed the study of macroalgal-associated microbial communities by enabling functional analysis beyond the fraction of microorganisms readily recovered using conventional cultivation. Rather than treating cultivation failure as evidence that most marine microorganisms are intrinsically “unculturable,” a more accurate interpretation is that standard laboratory media and incubation conditions recover only a biased subset of naturally occurring microbial diversity. Many host-associated microorganisms may depend on specific nutrients, signaling molecules, spatial associations, or physicochemical conditions that are difficult to reproduce outside the macroalgal surface environment [41,42].

Shotgun metagenomics circumvents part of this limitation by sequencing total community DNA recovered directly from host-associated microbial assemblages. It can reveal genes, metabolic pathways, population structure, and, when sequencing depth and community complexity permit, partial or near-complete genomes of uncultivated microorganisms. However, metagenomics primarily describes the genetic potential present within the community. It does not independently demonstrate that predicted genes are expressed, that encoded enzymes are active, or that the corresponding metabolic processes occur at ecologically meaningful rates [6].

This distinction is particularly important in macroalgal microbiomes, where microbial communities are often taxonomically uneven, spatially structured, and strongly influenced by host tissue, season, and environmental conditions. Functional interpretation therefore depends not only on detecting genes but on determining which organisms carry them, how reliably those genes have been reconstructed and annotated, and whether the predicted functions can subsequently be validated experimentally [6,7].

### 4.1. Beyond Cultivation: What Shotgun Metagenomics Adds

Traditional cultivation remains essential for experimental microbiology because isolated strains can be manipulated, physiologically characterized, and tested for direct effects on the host. Nevertheless, cultivation introduces substantial ecological bias. Rapidly growing, metabolically flexible organisms are generally easier to recover than microorganisms requiring host-derived compounds, microbial cross-feeding, specific attachment surfaces, or unusual nutrient conditions.

Shotgun metagenomics provides a complementary approach by accessing community DNA without requiring prior isolation [6]. In macroalgal systems, DNA can be extracted from surface-associated biofilms, dissected tissues, or whole holobiont preparations, depending on the biological question [43]. The term “metagenomic community DNA” is preferable in this context to “environmental DNA,” because the latter is widely used for DNA shed into environmental matrices and recovered for biodiversity detection rather than for direct genome-resolved characterization of host-associated communities [44].

After sequencing, metagenomic reads can be analyzed directly or assembled into longer contiguous sequences. Gene prediction and functional annotation can then identify coding sequences associated with transport, nutrient transformation, carbohydrate utilization, motility, signaling, secondary metabolism, and other microbial functions [6]. Comparison among hosts, tissues, seasons, or environments can reveal differences in the abundance and distribution of these functional capacities.

The principal advantage of this approach is therefore not that metagenomics directly describes microbial activity, but that it substantially expands the range of organisms and metabolic capabilities that can be investigated without cultivation.

Its limitations are equally important. Assembly efficiency depends on sequencing depth, genome abundance, strain heterogeneity, repeat content, and community complexity. Functional annotation is constrained by reference databases, while genes with weak similarity to characterized proteins may remain unassigned or be assigned only broad functional categories [45]. Consequently, metagenomics can uncover previously inaccessible biological potential while still leaving substantial portions of the community functionally unresolved.

### 4.2. Metagenome-Assembled Genomes and Taxon-Resolved Functional Ecology

One of the major advances enabled by shotgun metagenomics is the reconstruction of metagenome-assembled genomes (MAGs) [6,46]. Following assembly, contigs can be grouped into genomic bins using combinations of sequence composition and coverage patterns [47]. Quality assessment based on genome completeness and contamination can then identify bins suitable for downstream functional interpretation [48].

MAGs are particularly valuable because they connect metabolic genes to specific microbial populations [4,6,46]. Community-level metagenomic profiles may indicate that genes for vitamin biosynthesis, alginate degradation, chemotaxis, or nitrogen metabolism are abundant, but only genome-resolved analysis can begin to establish whether these capabilities are widely distributed across the community or concentrated within particular taxa.

The kelp *Nereocystis luetkeana* provides an informative example. Genome-resolved metagenomic analysis recovered 79 bacterial MAGs spanning several major bacterial lineages, including Pseudomonadota, Bacteroidota, Verrucomicrobiota, Planctomycetota, Bdellovibrionota, and Patescibacteria [4]. A subset of these populations was detected across different sampling periods and locations, suggesting that some bacterial associations recur despite substantial temporal and spatial variation in the host environment.

The study also demonstrated substantial genomic differentiation among kelp-associated populations of *Granulosicoccus*. Comparative analysis showed a relatively limited core gene repertoire accompanied by a large accessory component, consistent with considerable genomic plasticity among closely related populations [4]. Such variation is ecologically important because strains or species that appear taxonomically similar may differ substantially in substrate utilization, transport capacity, stress tolerance, or interactions with the host.

Kelp-associated *Granulosicoccus* MAGs encoded genes involved in motility, chemotaxis, type IV pilus formation, and extensive transport systems. These features are consistent with a surface-associated lifestyle in which microorganisms must locate host-derived chemical gradients, reach suitable tissues, attach to the thallus, and acquire available dissolved substrates [4].

Some MAGs also contained gene clusters associated with aerobic anoxygenic phototrophy, including bacteriochlorophyll synthesis and photosynthetic reaction-center components [4]. In the absence of canonical carbon-fixation pathways, such organisms are interpreted as photoheterotrophs capable of supplementing heterotrophic energy metabolism with light-derived energy. This represents an ecologically plausible adaptation to illuminated macroalgal surfaces, although genomic capacity alone does not establish the quantitative contribution of phototrophy to growth in situ.

Similarly, the identification of complete or near-complete pathways for vitamin biosynthesis, nitrogen transformations, polysaccharide utilization, and transport establishes the functional repertoire encoded by individual populations but does not demonstrate that those pathways are active under the sampled environmental conditions [4].

MAGs therefore provide an important intermediate level of biological resolution: they move interpretation beyond anonymous community genes by assigning metabolic capacities to reconstructed populations, but they remain genomic models rather than direct measurements of physiological activity [4].

### 4.3. Pangenomics and Functional Variation Within Host-Associated Populations

Genome reconstruction becomes particularly informative when multiple related MAGs or isolate genomes can be compared through pangenomic analysis [49,50]. A pangenome separates genes shared by most or all members of a lineage from accessory and strain-specific genes, allowing investigators to distinguish conserved biological features from locally variable adaptations.

In macroalgal microbiomes, this approach is useful because association with a host does not necessarily imply that all members of a bacterial genus perform equivalent functions. Closely related populations can differ in transport systems, carbohydrate-active enzymes, secondary metabolite pathways, stress responses, surface-attachment mechanisms, and other traits likely to influence their ecological role [51,52].

The *Granulosicoccus* populations associated with *Nereocystis luetkeana* illustrate this principle. Their relatively small shared gene repertoire and extensive accessory component indicate that taxonomic identity alone is insufficient to predict the complete functional contribution of a population to the kelp microbiome [4]. Some traits related to motility, host colonization, and environmental sensing appear broadly conserved, whereas other metabolic features vary among populations.

This distinction has wider implications for macroalgal microbiome ecology. Amplicon-based taxonomic surveys can reveal that the same genus occurs across different hosts or locations, but they cannot establish whether those populations possess the same functional repertoire. Conversely, taxonomically different communities may retain similar functional capacities through ecological redundancy [6,8].

Genome-resolved metagenomics and pangenomics therefore help address two related questions: which taxa carry particular functions, and how conserved are those functions within and among microbial lineages?

These questions become especially important when interpreting whole-community pathway profiles. A pathway enriched at the community level may reflect a broadly distributed ecological capability, or it may arise predominantly from one abundant population carrying many copies of the relevant genes [8]. Taxon-resolved genomic analysis is required to distinguish these alternatives.

### 4.4. Sequence-Based Metagenomic Mining

The genetic diversity recovered from macroalgal microbiomes also provides a substantial resource for biotechnology. One strategy for exploiting this diversity is sequence-based metagenomic mining, in which candidate genes and biosynthetic pathways are identified computationally using similarity to known protein families, conserved domains, genomic neighborhoods, and predictive models [41,53].

Profile hidden Markov models and curated databases can be used to identify candidate carbohydrate-active enzymes, transporters, sulfatases, and other protein families. For complex carbohydrates, platforms such as dbCAN facilitate prediction of carbohydrate-active enzyme families and associated gene clusters. Biosynthetic gene-cluster prediction tools such as antiSMASH can identify genomic regions potentially encoding non-ribosomal peptides, polyketides, terpenes, ribosomally synthesized and post-translationally modified peptides, and other specialized metabolites [54,55].

Sequence-based mining is efficient because large metagenomic datasets can be screened rapidly and candidate genes prioritized before experimental characterization [56]. Genome-resolved analysis further allows candidates to be linked to the microbial populations that encode them and can reveal whether biosynthetic pathways occur within persistent host-associated taxa or transient members of the community.

However, sequence-based discovery is inherently dependent on existing biological knowledge [56]. Genes that are highly divergent from characterized proteins may escape detection, whereas similarity to a known enzyme family does not guarantee identical substrate specificity or biochemical activity. Prediction of a biosynthetic gene cluster likewise identifies biosynthetic potential but does not demonstrate that the cluster is expressed or that the predicted metabolite is produced.

The correct endpoint of sequence-based mining is therefore a candidate, not a validated biocatalyst or natural product.

This distinction becomes especially important when metagenomic candidates are presented as industrially relevant enzymes. Sequence prediction can justify prioritization for cloning and testing, but properties such as catalytic efficiency, substrate range, thermostability, halotolerance, pH tolerance, secretion efficiency, and compatibility with industrial processes must be determined experimentally.

### 4.5. Function-Based Metagenomics

Function-based metagenomics offers a complementary strategy that does not depend primarily on sequence similarity. Community DNA is fragmented and cloned into suitable vectors, including plasmids, cosmids, fosmids, or bacterial artificial chromosomes, and introduced into a heterologous host. Clone libraries can then be screened directly for a phenotype or biochemical activity of interest [41,42].

For macroalgal biotechnology, relevant screens can include hydrolysis of alginate, agar, carrageenan, *Ulva*n, laminarin, or other polysaccharides; lipolytic or proteolytic activity; production of pigments; inhibition of microbial competitors; and other detectable phenotypes [41,42,57].

The major advantage of function-based screening is that activity can be discovered without requiring close sequence similarity to previously characterized proteins. This makes the approach particularly valuable for identifying novel catalytic mechanisms or enzyme families that may be poorly represented in current databases [57].

Its limitations are substantial. Environmental genes must be correctly transcribed and translated by the heterologous host, proteins must fold appropriately, cofactors must be available, multigene pathways must be expressed in the correct stoichiometry, and secreted or membrane-associated proteins may not be processed correctly. Many marine biosynthetic pathways therefore remain silent when introduced into standard laboratory hosts such as *Escherichia coli* [57].

Screening also favors activities that can be detected using robust laboratory assays. Complex ecological functions that require interactions among several microbial species, host-derived signals, unusual metabolites, or highly specific environmental conditions may be difficult to recover using conventional clone libraries [58].

Sequence-based and function-based strategies should consequently be regarded as complementary rather than competing approaches. Sequence-based mining provides scale and genomic context; functional screening provides direct evidence of biological activity. Combining both approaches can substantially improve candidate prioritization and validation (Table 2).

### 4.6. From Gene Discovery to Functional Validation

Metagenomic discovery should be regarded as the beginning rather than the endpoint of a bioprospecting pipeline. A candidate gene identified computationally must pass through successive levels of validation before it can reasonably be considered a useful biocatalyst or biotechnology lead [59].

Initial validation should confirm coding-sequence integrity, taxonomic context, conserved domains, and genomic neighborhood. Candidate genes can then be cloned or synthesized and expressed in an appropriate heterologous system. Biochemical characterization is subsequently required to establish substrate specificity, catalytic properties, temperature and pH optima, salt tolerance, cofactor requirements, and kinetic parameters. For biosynthetic gene clusters, expression must be linked directly to production and chemical identification of the corresponding metabolite [59].

Technological relevance requires an additional level of assessment, including expression yield, stability, scalability, substrate availability, process compatibility, production cost, and regulatory constraints [59]. This progression can be summarized as:

Metagenomic detection → genomic assignment → functional prediction → heterologous expression → biochemical validation → process evaluation → technological application.

Metagenomic discovery therefore identifies candidates; experimental and process-level validation determine whether those candidates have practical value.

### 4.7. Marine Metagenomic Precedents and Their Relevance to Macroalgal Microbiomes

Some of the best-known examples illustrating the power of marine metagenomics originate outside macroalgal systems. The elucidation of patellamide biosynthesis in the cyanobacterial symbiont *Prochloron didemni*, associated with the tunicate *Lissoclinum patella*, demonstrated how genomic and heterologous-expression approaches can reveal the microbial origin and biosynthetic machinery of a natural product previously recovered from a complex marine association [42,60].

This example is informative as a marine metagenomic precedent, but it should not be treated as direct evidence for natural-product discovery from macroalgal microbiomes. Its relevance lies in demonstrating a general strategy: uncultivated or difficult-to-culture symbionts can contain biosynthetic pathways that become accessible through culture-independent genome reconstruction and subsequent experimental expression [53,60].

The same example also illustrates a more specific methodological problem relevant throughout this review: biosynthetic attribution requires linking a metabolite to a microbial genome, strain, or experimentally expressed pathway, rather than assuming host origin from extraction source alone. Natural products recovered from a complex marine association cannot automatically be assigned to the visible host organism; the patellamide case demonstrates this principle directly, since the compounds were initially associated with the tunicate host before genomic and heterologous-expression work reassigned their biosynthetic origin to the cyanobacterial symbiont. This methodological lesson applies directly to macroalgal systems, which provide an attractive context for integrated genome–metabolite discovery, but where the number of examples in which a metagenomically predicted BGC has been carried through to complete structural and functional validation remains limited (Section 6.4).

Macroalgal microbiomes provide a similarly complex discovery environment, but the number of examples in which a metagenomically predicted pathway has been taken through complete biochemical validation and product characterization remains comparatively limited. This gap should be considered an opportunity rather than evidence that predicted macroalgal microbiome functions have already been translated successfully at scale.

The most mature route forward is therefore one that combines ecological context with genome-resolved discovery and experimental validation. Host association can help identify microbial populations adapted to unusual substrates or environmental conditions, metagenomics can reveal their encoded metabolic potential, and cultivation or heterologous expression can test whether those functions are technologically useful.

### 4.8. Methodological Limits and the Need for Multi-Omics Integration

Metagenomics greatly expands access to microbial functional diversity, but several limitations constrain ecological interpretation. First, DNA abundance does not indicate biological activity. Genes may be present but weakly expressed, inactive under sampled conditions, or relevant only under environmental states not captured during sampling. Second, pathway abundance can be strongly influenced by community composition. A highly abundant microbial population may contribute disproportionately to pathway-associated genes and create an apparent whole-community difference. Third, genome reconstruction is imperfect. MAGs may remain incomplete because of limited sequencing depth, strain heterogeneity, assembly fragmentation, or binning uncertainty, and apparent pathway absence should therefore be interpreted cautiously. Finally, functional annotation remains probabilistic, particularly for poorly characterized protein families. Pathway reconstruction can consequently imply greater mechanistic precision than the underlying gene assignments justify [6,7].

These limitations make complementary approaches essential. Metatranscriptomics can establish transcription, metaproteomics can confirm protein production, metabolomics can identify associated chemical products, stable-isotope approaches can trace substrate transformation, and cultivation or synthetic-community experiments can test causality (paralleling the evidence hierarchy in Figure 1) [7]. Spatial resolution is also increasingly important because macroalgal thalli contain heterogeneous tissues and microscale environments that may be obscured in whole-thallus metagenomes.

Metagenomics is therefore most powerful as a framework for generating taxon-resolved and experimentally testable hypotheses rather than as a standalone measure of ecological function.

### 4.9. From Community Profiles to Taxon-Resolved Functional Interpretation

Whole-community functional profiles reveal which metabolic capacities are represented in a microbiome, but they do not identify how those functions are distributed among microbial populations. Taxonomic attribution adds a second level of interpretation by asking which organisms encode particular pathways and whether the corresponding signal is broadly distributed, concentrated within a functional guild, or dominated by a single abundant population [6,8].

These alternatives have different ecological implications. Broadly distributed functions may represent relatively stable community properties, whereas functions concentrated within one or a few taxa identify more specific ecological actors and potential targets for cultivation, genome mining, or bioprospecting.

Experimental and multi-omic approaches then address a further question: whether those taxon-attributed functions are expressed and ecologically consequential.

Taxon-resolved analysis therefore provides an essential bridge between community-level pathway abundance and mechanistic interpretation. This distinction becomes particularly important later in this review, where comparative functional profiles of invasive macroalgal holobionts are examined in relation to dominant microbial populations.

## 5. Glycan-Degrading Systems in Macroalgal Microbiomes: CAZymes, PULs, and Functional Specialization

Macroalgal cell walls contain structurally diverse polysaccharides that differ substantially among brown, green, and red algal lineages. These polymers represent important sources of carbon and energy for associated microorganisms, but their chemical complexity requires specialized enzymatic systems for recognition, deconstruction, transport, and further metabolism. As a result, macroalgal surfaces and decaying tissues select for microbial populations with distinct repertoires of carbohydrate-active enzymes and polysaccharide-utilization systems [61,62,63,64,65].

Metagenomic and genomic studies have revealed that many macroalgal-associated bacteria, particularly members of the Bacteroidota, encode large and diverse suites of carbohydrate-active enzymes (CAZymes) and, in some cases, polysaccharide utilization loci (PULs). These genomic features provide strong evidence for adaptation to complex algal glycans. However, as with other metagenomically inferred functions, the presence of a CAZyme family or a predicted PUL does not by itself establish substrate specificity, expression, enzymatic activity, or ecological importance in situ. Functional interpretation therefore requires careful distinction between predicted glycan-degrading capacity and experimentally demonstrated substrate utilization [61,64].

### 5.1. Macroalgal Glycans as Ecological and Biochemical Substrates

The major macroalgal lineages differ markedly in cell-wall chemistry. Brown macroalgae contain alginate as a major structural polymer, together with sulfated fucose-rich polysaccharides collectively referred to as fucoidans. Laminarin, although not a structural cell-wall polymer, serves as an important storage glucan in many brown algae. Green macroalgae contain lineage-specific polysaccharides including *Ulva*ns, whereas red algae are characterized by sulfated galactans such as agarans and carrageenans [61,62,63].

These polysaccharides differ in monosaccharide composition, linkage type, branching, uronic acid content, and degree and position of sulfation. Their degradation therefore requires combinations of hydrolytic, eliminative, de-esterifying, and desulfating enzymes rather than a single universal carbohydrate-degradation pathway [61,63].

Carbohydrate-active enzymes are commonly classified into several functional groups, including glycoside hydrolases (GHs), polysaccharide lyases (PLs), carbohydrate esterases (CEs), glycosyltransferases, and carbohydrate-binding modules. In the context of macroalgal biomass utilization, GHs and PLs are especially important for cleavage of glycosidic or uronic-acid-containing backbones, while sulfatases and esterases can remove substituents that otherwise restrict enzyme access [55].

The ecological significance of these enzyme systems extends beyond decomposition of senescent algal biomass. Soluble oligosaccharides, storage polysaccharides, mucus-associated glycans, and surface polymers can all provide substrates for epiphytic and surrounding heterotrophic microorganisms. Nevertheless, the relative importance of intact cell-wall degradation versus utilization of soluble or partially degraded material varies among taxa and ecological conditions and should not be inferred solely from the presence of CAZyme genes [61].

### 5.2. Bacteroidota as Prominent Glycan-Utilizing Members of Macroalgal Microbiomes

Members of the phylum Bacteroidota are repeatedly associated with the degradation of complex marine polysaccharides and are common components of many macroalgal microbiomes. Families such as Flavobacteriaceae and related lineages contain organisms with extensive repertoires of glycoside hydrolases, polysaccharide lyases, sulfatases, and transport systems, consistent with adaptation to polymer-rich marine habitats [61,64].

Comparative genomic analyses of bacteria associated with co-located green, brown, and red macroalgae have shown that some host-associated Bacteroidota contain larger numbers of predicted CAZymes and PULs than related planktonic populations [64]. Such enrichment supports the hypothesis that macroalgal surfaces represent selective environments favoring microorganisms capable of utilizing complex host-associated glycans.

However, this pattern should not be interpreted as universal across all Bacteroidota. Closely related bacterial lineages can differ substantially in their carbohydrate-degrading repertoires, and some host-associated taxa possess few or no recognizable PULs. This heterogeneity suggests a division of labor within the microbial community, in which some populations act as primary degraders of complex polymers while others consume smaller oligosaccharides or metabolites released during degradation [61,64].

The resulting community structure may therefore involve both direct polymer degraders and organisms participating through metabolic cross-feeding. Such functional differentiation is consistent with the broader principle that taxonomic membership alone does not determine ecological function [61].

### 5.3. Polysaccharide Utilization Loci as Coordinated Glycan-Degradation Systems

In many Bacteroidota, carbohydrate utilization is organized within polysaccharide utilization loci. A PUL generally consists of physically clustered genes encoding proteins involved in substrate recognition, binding, enzymatic processing, transport, and regulation [55,61].

Canonical PULs frequently include a SusC-like TonB-dependent outer-membrane transporter together with a SusD-like substrate-binding protein. Additional genes may encode glycoside hydrolases, polysaccharide lyases, carbohydrate esterases, sulfatases, transcriptional regulators, and other components required for the processing of a particular carbohydrate [61].

This genomic organization provides an efficient mechanism for coordinating glycan recognition and utilization. In marine bacteria, PUL architectures can be particularly complex because many algal polysaccharides are sulfated or contain uronic acids and require several sequential enzymatic reactions before their constituent sugars can be transported and metabolized [61,63].

The presence of a PUL can therefore provide stronger functional context than the detection of an isolated CAZyme gene. Even so, assignment of a PUL to a specific substrate remains a prediction unless supported by biochemical characterization, transcriptomic induction, growth on defined substrates, gene disruption, or other experimental evidence [55,61].

This distinction is particularly important for marine polysaccharides because structurally related substrates may be processed by overlapping enzyme families, and individual CAZyme families can contain proteins with different substrate specificities [55,61].

### 5.4. Functional Specialization Across Major Macroalgal Glycan Classes

#### 5.4.1. Alginate

Alginate is a major structural polysaccharide of brown macroalgae and consists of varying proportions of β-D-mannuronate and α-L-guluronate residues. Its degradation is primarily mediated by alginate lyases belonging to several polysaccharide-lyase families [4,61].

Metagenomic analysis of the *Nereocystis luetkeana* microbiome identified genes annotated as alginate lyases in multiple bacterial lineages, including members of Saprospiraceae, Maricaulaceae, and Granulosicoccaceae [4]. These findings indicate that the capacity for alginate utilization is distributed across more than one taxonomic group within the kelp-associated community.

The presence of alginate-lyase genes is consistent with adaptation to a brown-algal environment but does not necessarily imply degradation of intact healthy host tissue. Alginate may become available through mucus, tissue turnover, mechanical damage, senescence, or degradation of detached biomass. The ecological context in which individual populations deploy these enzymes therefore remains important [4,61].

#### 5.4.2. Laminarin

Laminarin is a β-glucan storage polysaccharide found in many brown algae and can represent an important carbon source for marine heterotrophic bacteria. Its degradation typically requires β-1,3-glucanases and enzymes capable of processing β-1,6-linked branches [61].

Numerous marine Bacteroidota possess laminarin-degrading systems, although their distribution differs among taxa and environments. The absence of recognizable laminarinase genes from particular MAGs should not be interpreted as evidence that an entire macroalgal microbiome lacks the capacity to utilize laminarin, because incomplete genome reconstruction, transient community members, and alternative enzyme families can all affect detection [61].

Similarly, the presence of candidate GH families associated with β-glucan degradation requires biochemical confirmation before precise substrate specificity can be assigned.

#### 5.4.3. Ulvan

*Ulva*ns are sulfated polysaccharides characteristic of the cell walls of several green macroalgae, particularly *Ulva*. Their structural complexity requires combinations of *Ulva*n lyases, glycoside hydrolases, sulfatases, and other accessory enzymes [62].

Bacterial genera, including *Zobellia* and *Maribacter*, contain genomic systems associated with utilization of green-algal polysaccharides, and several polysaccharide-lyase families have been linked to *Ulva*n degradation. Such systems illustrate how host-associated bacteria can evolve highly specialized enzyme repertoires targeting lineage-specific macroalgal substrates [62,64].

Because *Ulva*n structure varies among species and environmental conditions, however, functional assignment should ideally be supported by growth experiments or biochemical characterization of individual enzymes rather than inferred solely from CAZyme-family membership [62].

#### 5.4.4. Fucoidans and Other Sulfated Brown-Algal Polysaccharides

Fucoidans are sulfated, fucose-rich polysaccharides characteristic of brown algae. Their structural variability and sulfation contribute to substantial substrate complexity [4,64].

Macroalgal-associated Bacteroidota and Verrucomicrobiota can possess large repertoires of sulfatases and other carbohydrate-processing enzymes. In the *Nereocystis* microbiome, members of the Akkermansiaceae contained particularly extensive sulfatase repertoires [4]. This genomic architecture is consistent with adaptation to sulfated polysaccharides.

The precise substrates of individual sulfatases cannot, however, be inferred confidently from gene number alone. Large sulfatase repertoires indicate substantial potential for processing sulfated molecules but may encompass multiple substrates beyond fucoidans [4].

#### 5.4.5. Red-Algal Galactans

Agarans and carrageenans are sulfated galactans characteristic of many red algae and represent additional substrates requiring specialized microbial enzyme systems. Agarases and carrageenases have been characterized from several marine bacteria, including taxa associated with algal surfaces and decomposing biomass [63,65].

These enzymes are of considerable interest because they can generate oligosaccharides with properties distinct from the parent polymers. Nevertheless, as with other CAZymes, the presence of predicted agarase or carrageenase genes in metagenomic data should be treated as evidence of potential until substrate specificity and catalytic activity are confirmed experimentally [63,65] (Table 3).

### 5.5. Glycan-Degradation Networks and Microbial Division of Labor

Complex polysaccharide degradation is rarely the activity of a single enzyme and may not be the activity of a single microbial population. Breakdown of sulfated algal glycans can require extracellular or surface-associated cleavage, removal of sulfate or acetyl groups, transport of oligosaccharides, intracellular hydrolysis, and subsequent catabolism of released monosaccharides [61,63,65].

This creates opportunities for metabolic division of labor.

Primary degraders may possess the extracellular enzymes and transport systems required to access intact polymers, whereas secondary consumers may lack those systems but efficiently utilize oligosaccharides or monomers released by neighboring organisms. Other community members may specialize in downstream fermentation products, organic acids, or other metabolites generated during glycan turnover [61].

Such cross-feeding can make the functional structure of the community more complex than predicted from the genome of any single taxon. A microorganism lacking a complete PUL may still participate actively in algal-carbon utilization if it depends on degradation products generated by another population [61].

Consequently, absence of a particular CAZyme repertoire should not automatically be interpreted as ecological irrelevance to carbohydrate metabolism. It may instead indicate a different position within a community-level trophic network.

This perspective also reinforces the importance of taxon-resolved analysis. Whole-community CAZyme abundance can reveal the overall carbohydrate-processing potential of a microbiome, but it cannot show whether those functions are concentrated in a few specialized degraders or distributed broadly among community members [61,64].

### 5.6. From CAZyme Prediction to Demonstrated Substrate Utilization

CAZyme annotation is one of the most powerful approaches for identifying candidate carbohydrate-processing functions in metagenomes, but it also illustrates the limitations of sequence-based functional prediction—an instance of the general genomic-potential-versus-demonstrated-function distinction shown in Figure 1 [55,61].

A predicted CAZyme family assignment establishes structural or evolutionary similarity to characterized proteins. It does not automatically establish the exact substrate, catalytic efficiency, environmental activity, or physiological role of the encoded enzyme [55,61].

Stronger functional evidence can be obtained through several complementary approaches. Growth experiments using defined polysaccharides can establish whether an organism is capable of utilizing a substrate. Transcriptomic or proteomic measurements can determine whether candidate genes are induced during substrate exposure. Recombinant expression and purified-enzyme assays can confirm catalytic activity and substrate specificity. Gene deletion or disruption can demonstrate whether a particular enzyme is necessary for utilization of the polymer [55,61].

For complete PULs, coordinated transcription in response to a defined glycan provides especially strong evidence linking genomic organization to substrate utilization [61].

An evidence hierarchy for glycan-degrading systems can therefore be represented as:

CAZyme-family prediction → predicted PUL → substrate-dependent expression → demonstrated growth or degradation → purified-enzyme characterization → experimentally resolved pathway.

This progression should be reflected explicitly when discussing the biotechnological significance of candidate enzymes.

### 5.7. Biotechnological Potential of Macroalgal Microbiome CAZymes

Macroalgal-associated microorganisms are attractive sources of enzymes because they naturally encounter substrates and physicochemical conditions relevant to marine biomass processing. Candidate enzymes may therefore possess properties such as salt tolerance, activity at moderate temperatures, or specificity toward polysaccharides that are uncommon in terrestrial biomass [41,42].

Alginate lyases, *Ulva*n lyases, agarases, carrageenases, β-glucanases, sulfatases, and related enzymes have potential applications in the conversion of macroalgal biomass into oligosaccharides, fermentable sugars, fine chemicals, and other value-added products [61,62,63,64,65].

However, metagenomic identification alone does not establish industrial usefulness. A candidate enzyme must first be expressed and biochemically characterized. Relevant parameters include substrate specificity, catalytic efficiency, temperature and pH optima, salt tolerance, enzyme stability, product profile, recombinant yield, and compatibility with downstream processes.

This distinction is particularly important when environmental sequences are described as “green biocatalysts” or industrial enzymes before their properties have been determined. Metagenomics identifies biocatalyst candidates; industrial relevance emerges only after experimental and process-level validation.

Sequence-based discovery and functional screening can be combined to accelerate this transition. Genome mining can identify candidate enzyme families and their taxonomic sources, while activity-based screening can reveal biochemical functions that are poorly represented in current databases. Subsequent recombinant expression and enzymology can then establish whether those candidates have practical value.

### 5.8. Host Glycan Chemistry as a Selective Force on Microbiome Function

The recurrent enrichment of glycan-degrading systems in macroalgal-associated bacteria raises a broader ecological question: to what extent does host polysaccharide chemistry structure microbiome functional composition?

Green, brown, and red algae offer substantially different carbohydrate environments. It is therefore reasonable to expect that host identity can select for microbial populations possessing different carbohydrate-processing repertoires. Comparative studies of bacteria associated with co-located macroalgal hosts support this possibility by identifying variation in CAZyme and PUL content among host-associated communities [64].

However, host chemistry is unlikely to be the only determinant. Microbial phylogeny strongly constrains enzyme repertoires, while season, tissue age, environmental nutrient availability, host condition, and microbial dispersal can all influence which glycan-utilizing populations become established.

Functional convergence may also occur. Different microbial taxa can encode enzymes capable of processing similar substrates, meaning that host-specific carbohydrate utilization may be maintained despite taxonomic turnover.

The emerging picture is therefore not one of simple one-host/one-degrader specificity, but of ecological filtering acting on partially redundant functional repertoires. Identifying how host glycan composition, microbial phylogeny, and environmental conditions interact to structure these repertoires remains an important challenge for macroalgal microbiome ecology.

### 5.9. From Glycan Ecology to Genome-Guided Bioprospecting

Glycan-degrading systems provide a useful bridge between ecological microbiome research and biotechnology. In ecological terms, CAZymes and PULs reveal how microbial populations access one of the most abundant resource pools associated with macroalgae. In biotechnological terms, the same systems provide candidate enzymes for controlled deconstruction of marine biomass.

The crucial methodological principle is that these two interpretations require different levels of evidence.

A CAZyme detected in a metagenome may indicate ecological potential. A substrate-responsive PUL provides stronger evidence of a specific ecological function. A recombinantly expressed enzyme with demonstrated activity establishes biochemical function. Only subsequent process-level testing can determine whether that activity is technologically useful.

Maintaining these distinctions avoids converting genomic prediction directly into commercial promise and allows macroalgal microbiome bioprospecting to proceed through a transparent sequence of discovery, validation, and application.

The same principle becomes even more important for the natural-product pathways considered in the following section, where biosynthetic gene clusters can reveal substantial chemical potential but cannot establish metabolite production without experimental validation.

## 6. Natural Product Discovery and Translational Potential of Macroalgal Microbiomes

Macroalgal-associated microorganisms occupy chemically competitive environments in which access to space, nutrients, and host-derived substrates is strongly contested. These interactions can favor the production of secondary metabolites, signaling molecules, extracellular polymers, and other compounds with ecological functions that may also have biotechnological value. As a result, seaweed-associated bacteria have attracted increasing attention as sources of antimicrobial compounds, biosurfactants, pigments, signaling molecules, and biosynthetic pathways that would not be identified by analyzing the algal host alone [66].

However, the translational potential of these systems must be interpreted cautiously. Detection of a biosynthetic gene cluster does not establish metabolite production. Isolation of a metabolite from a seaweed-associated bacterium does not demonstrate that the compound contributes to host fitness in nature. Similarly, antimicrobial activity observed in vitro does not automatically translate into therapeutic, agricultural, or industrial utility.

A useful distinction is therefore required between four progressively stronger levels of evidence:

Biosynthetic potential → metabolite production → validated biological activity → demonstrated application.

This section considers natural-product discovery and biotechnology within that framework.

### 6.1. Biosynthetic Gene Clusters as Indicators of Chemical Potential

Microbial secondary metabolites are frequently encoded by biosynthetic gene clusters (BGCs), in which multiple genes involved in precursor synthesis, enzymatic modification, transport, regulation, and resistance occur within a localized genomic region. Major BGC classes include non-ribosomal peptide synthetases, polyketide synthases, ribosomally synthesized and post-translationally modified peptides, and terpene-associated pathways [54].

Shotgun metagenomics and genome-resolved sequencing provide access to such pathways without requiring prior cultivation of the producing organism. Computational tools including antiSMASH can identify genomic regions with architectures resembling characterized BGCs, allowing microbial populations with high biosynthetic potential to be prioritized for further study [54].

This approach is particularly relevant to macroalgal microbiomes because many associated microorganisms are difficult to recover using conventional laboratory media, while secondary metabolite pathways may remain transcriptionally silent under standard culture conditions. Sequence-based mining therefore expands access to biosynthetic diversity that could otherwise remain undetected.

Nevertheless, BGC prediction remains an inference. A computationally identified cluster indicates that an organism possesses genes consistent with a particular class of biosynthetic machinery. It does not demonstrate that the cluster is expressed, that a metabolite is produced, or that the chemical structure can be predicted accurately from sequence alone.

The term “silent BGC” should therefore be used carefully. In some cases, silence is demonstrated experimentally by comparing genomic presence with lack of expression under defined culture conditions. In other cases, the cluster is merely predicted but has not been examined transcriptionally. These situations are not equivalent [53,56].

Genome mining is consequently most useful as a candidate-discovery and prioritization tool, particularly when combined with metatranscriptomics, metabolomics, heterologous expression, or cultivation approaches capable of linking a genomic cluster to a chemical product [53,56].

### 6.2. Seaweed-Associated Bacteria as Sources of Bioactive Metabolites

Several bioactive compounds have been isolated directly from bacteria associated with macroalgae, providing stronger evidence than sequence prediction alone.

Kocumarin, identified from *Kocuria marina* associated with the brown alga *Pelvetia canaliculata*, has been reported to exhibit antimicrobial activity against selected bacterial and fungal targets [67]. Likewise, decylprodigiosin was isolated from a *Streptomyces violaceoruber* strain associated with the green alga *Codium tomentosum* and characterized as a member of the prodigiosin family with antibacterial and cytotoxic properties [68].

These examples demonstrate that macroalgal-associated bacteria can produce chemically distinctive metabolites with measurable biological activity.

They do not, however, support the broader claim that many therapeutically relevant compounds historically attributed to macroalgae are actually produced by their microbiota. Such a generalization requires direct biosynthetic attribution on a compound-by-compound basis.

A more plausible conclusion is that macroalgal-associated microorganisms constitute an additional chemical reservoir that may contribute metabolites distinct from those synthesized by the algal host. This distinction is important in biodiscovery studies because extraction of whole algal material can obscure the biological origin of individual compounds.

Establishing microbial origin requires stronger evidence, such as isolation of the producing strain, identification and expression of the relevant BGC, isotope incorporation, spatial metabolite localization, or other approaches linking a compound to a defined microbial source.

### 6.3. Marine Metagenomic Precedents and the Problem of Biosynthetic Attribution

The patellamide/*Prochloron didemni* precedent and its implications for biosynthetic attribution in macroalgal systems are discussed in Section 4.7. In brief: natural products recovered from a complex marine association cannot automatically be assigned to the visible host organism, and the same logic applies to seaweed-associated communities, where biosynthetic attribution requires linking a metabolite to a microbial genome, strain, or experimentally expressed pathway rather than assuming host origin from extraction source alone. Macroalgal systems therefore provide an attractive context for integrated genome–metabolite discovery, but the number of examples in which a metagenomically predicted BGC has been carried through to complete structural and functional validation remains limited.

### 6.4. From Genome Mining to Chemical Validation

The transition from predicted BGC to validated natural product requires several distinct steps. First, the genomic integrity of the candidate cluster must be confirmed. This includes determining whether the complete biosynthetic machinery has been assembled, whether the genes belong to the same microbial population, and whether the cluster contains recognizable regulatory and transport components.

Second, evidence of expression is required. Metatranscriptomic or transcriptomic analysis can determine whether the cluster is transcriptionally active under natural or experimentally manipulated conditions. Proteomic analysis can provide additional support for pathway activity.

Third, the corresponding metabolite must be detected chemically. Untargeted or targeted metabolomics, high-resolution mass spectrometry, and chromatographic separation can be used to associate particular compounds with the presence or expression of the candidate BGC [53,56].

Fourth, the relationship between genotype and metabolite should be established experimentally. This may involve cultivation of the producing organism, heterologous expression of the cluster, gene disruption, or comparison among strains that differ in cluster presence.

Only after this connection has been demonstrated can a predicted BGC reasonably be described as encoding a characterized natural product pathway [53,56]. This distinction is especially important in metagenomic datasets because structurally complex BGCs can be fragmented across contigs, incorrectly binned, or incompletely reconstructed. Large multidomain enzymes such as polyketide synthases and non-ribosomal peptide synthetases are particularly challenging to assemble accurately from complex communities.

### 6.5. Context-Dependent Expression and the OSMAC Principle

Secondary metabolite production is strongly influenced by environmental context. Microbial strains can produce substantially different chemical profiles depending on nutrients, salinity, temperature, oxygen availability, cell density, neighboring microorganisms, and other environmental cues. This principle is commonly described as “One Strain Many Compounds” (OSMAC), reflecting the ability of a single microbial genotype to alter secondary metabolite production under different cultivation conditions [69].

For macroalgal-associated microorganisms, this context dependency is particularly relevant because the natural thallus environment may contain host-derived metabolites, microbial signaling molecules, surface-associated gradients, and interspecies interactions that are absent from standard laboratory cultures. Consequently, biosynthetic pathways active in situ may be weakly expressed or silent after isolation. Conversely, laboratory stress or altered nutrient conditions may induce compounds that are not produced at substantial levels on the host.

Therefore, recovery of a microbial strain does not guarantee reproduction of its natural metabolome, and the absence of a metabolite under one culture condition does not establish that its biosynthetic pathway is inactive in nature.

Experimental strategies such as media diversification, co-culture, addition of host-derived metabolites, variation in salinity or nutrient conditions, and targeted manipulation of pathway regulators can help explore the chemical repertoire of seaweed-associated isolates.

The OSMAC framework is therefore valuable as a discovery strategy, but it also reinforces the need to distinguish biosynthetic capacity from ecologically realized chemistry.

### 6.6. Seaweed-Associated Microorganisms in Agricultural Biotechnology

Macroalgal-associated microorganisms have also been proposed as sources of compounds relevant to agriculture, including plant-growth regulators, defense elicitors, antimicrobial metabolites, extracellular polymers, and quorum-signaling molecules [66].

Some seaweed-associated bacteria produce metabolites with activities that can influence plant growth or microbial antagonism. Auxin-like and cytokinin-like effects associated with macroalgal bacterial partners provide one conceptual connection between microbial signaling and plant developmental biology. However, compounds involved in seaweed morphogenesis should not automatically be assumed to produce equivalent responses in terrestrial crops.

Similarly, N-acyl homoserine lactones (AHLs) and other microbial signaling molecules can influence plant defense pathways in terrestrial systems. The macroalgal microbiome may therefore represent a source of microorganisms capable of producing chemically relevant signaling molecules, but evidence that particular seaweed-associated strains can reliably enhance crop resistance under agronomic conditions remains more limited than the general literature on plant-associated bacteria [66,70].

Alginate-derived oligosaccharides and chitin-derived fragments have also been investigated as plant defense elicitors. In the context of macroalgal microbiomes, bacterial degradation of algal polymers could generate such compounds, but their formation in situ, ecological concentration, and subsequent applicability to terrestrial agriculture must be demonstrated rather than inferred from the presence of degradative enzymes [66].

The most likely interpretation is therefore that macroalgal microbiomes contain microbial functions and metabolites with potential agricultural relevance, but their translational maturity varies considerably among compound classes.

### 6.7. Antimicrobial and Biocontrol Potential

Microorganisms associated with macroalgae are exposed to intense competition for surface space and resources, providing ecological conditions in which antimicrobial or interference mechanisms may be advantageous.

Several associated bacteria have demonstrated inhibition of other microorganisms in laboratory assays. Such activities can arise from diffusible antibiotics, lipopeptides, bacteriocins, pigments, biosurfactants, quorum-interference mechanisms, or combinations of these factors.

For example, the manuscript identifies antimicrobial activity associated with *Kocuria marina* and *Streptomyces violaceoruber*, while other seaweed-associated strains, including *Bacillus halotolerans*, have been investigated for antagonism toward fungal plant pathogens [67,68,71].

These results justify continued screening of macroalgal-associated microorganisms for antimicrobial activity.

However, inhibition zones, minimum inhibitory concentrations, or antagonism in dual-culture assays represent early stages of validation. They do not demonstrate therapeutic efficacy, environmental persistence, crop protection, toxicity profile, or commercial viability [59,66].

Biocontrol development requires additional evidence, including identification of the active compound, reproducibility across target strains, dose–response characterization, toxicity testing, stability, performance under realistic environmental conditions, and, for agricultural applications, appropriate greenhouse or field validation [59,66]. The same caution applies to claims of anticancer activity. Cytotoxicity against cultured tumor cells identifies a potential lead, not an antineoplastic therapeutic.

### 6.8. Synthetic Biology and Heterologous Expression

One route to overcoming cultivation and production limitations is heterologous expression of candidate biosynthetic pathways in more tractable microbial hosts [53].

BGCs identified by genome mining can, in principle, be cloned or reconstructed and expressed in organisms such as *Escherichia coli*, *Bacillus subtilis*, or *Streptomyces* species. This strategy can provide access to metabolites from microorganisms that grow poorly, produce low compound yields, or fail to express the relevant pathway under laboratory conditions [53]. The approach is particularly attractive for complex natural products because it separates biosynthetic discovery from large-scale cultivation of the original marine organism.

However, heterologous expression remains technically demanding. BGCs can be large, transcriptional regulation may differ between donor and host, precursor availability may be limiting, enzymes may require specific cofactors, and membrane-associated or post-translational processes may not be reproduced correctly [57]. The successful identification of a BGC therefore does not imply that it can be transferred readily into an industrial chassis. Synthetic biology should instead be viewed as one stage within a broader pipeline:

BGC prediction → cluster validation → expression testing → metabolite identification → pathway optimization → production engineering.

Only after these steps can production scalability be evaluated realistically (Table 4).

### 6.9. From Discovery to Translation: A Readiness Framework

The biotechnological potential of macroalgal microbiomes is substantial, but the field benefits from distinguishing discovery from translation.

A practical evidence ladder, paralleling the general evidence hierarchy in Figure 1, can be defined as follows:

Stage 1—Genomic discovery: A candidate enzyme, pathway, or BGC is identified computationally.

Stage 2—Functional validation: The gene or pathway is expressed, and the predicted activity or metabolite is confirmed.

Stage 3—Biological validation: The compound or enzyme produces a reproducible effect in an appropriate experimental system.

Stage 4—Process validation: Production yield, stability, formulation, scalability, and process compatibility are assessed.

Stage 5—Application validation: Performance is demonstrated under conditions relevant to the intended industrial, agricultural, or biomedical use.

Most examples discussed in this review currently lie within the first three stages.

This should not be interpreted as a weakness. It reflects the current maturity of the field and identifies where future research effort is most needed. Presenting early genomic or biochemical discoveries as established industrial solutions would obscure these translational gaps rather than strengthen the case for continued investment.

### 6.10. A More Realistic View of the Macroalgal Microbiome as a Bioprospecting Resource

Macroalgal microbiomes represent attractive reservoirs of microbial diversity because their members are exposed to unusual polysaccharides, fluctuating salinity, strong competition for surface space, oxidative conditions, and chemically complex host environments. These selective pressures can favor metabolic capabilities with potential value in biotechnology.

The most promising strategy is not to assume that every predicted pathway constitutes a useful product, but to use ecological information to improve bioprospecting efficiency.

A microbial population repeatedly associated with a sulfated-polysaccharide-rich host may be prioritized for carbohydrate-active enzymes. A surface-associated competitor may be screened for antimicrobial or quorum-interference compounds. A strain adapted to strong osmotic fluctuations may contain enzymes or metabolites with useful stability properties. Genome-resolved metagenomics can then identify candidate functions, while cultivation, heterologous expression, metabolomics, and biochemical characterization establish whether those candidates have practical value.

In this framework, ecology provides the selection logic, metagenomics provides the discovery platform, and experimental validation determines the biotechnological reality.

This approach is more defensible than treating the macroalgal microbiome simply as an untapped catalog of valuable genes. It also provides a direct bridge to the ecological comparisons considered later in this review, where differences among host lifestyles may identify distinct microbial functions and taxa as rational targets for bioprospecting.

## 7. Generalizable Principles Beyond Macroalgae

Macroalgal microbiomes form part of a wider spectrum of algal–microbial associations that includes unicellular microalgae, cyanobacteria, and other photosynthetic microorganisms. Across these systems, microorganisms can influence nutrient acquisition, vitamin availability, metabolite exchange, stress responses, biomass productivity, and community stability. However, similarities in ecological function do not imply mechanistic equivalence among fundamentally different host systems.

Macroalgae differ from planktonic microalgae and cyanobacteria in several important respects. They possess persistent multicellular tissues, chemically differentiated surfaces, complex cell-wall polysaccharides, spatially structured biofilms, and life histories that can extend across seasons or years. Their associated microorganisms therefore occupy physical and chemical habitats that differ substantially from the transient diffusive environments surrounding individual planktonic cells.

Comparative analysis is nevertheless useful because it reveals which principles of algal–microbial interaction appear repeatedly across host types and which may represent particular features of macroalgal holobionts. The comparison is most informative when assessing evidence at equivalent levels: community association should be compared with community association, experimentally demonstrated exchange with experimentally demonstrated exchange, and genomic potential with genomic potential.

### 7.1. Shared Metabolic Principles Across Algal–Microbial Systems

One of the most recurrent features of algal–microbial associations is metabolic complementarity. Photosynthetic hosts release organic carbon compounds that can support heterotrophic microorganisms, while associated bacteria may provide vitamins, transform nutrients, produce signaling molecules, or alter the chemical environment surrounding the host.

In microalgal systems, bacterial interactions influence changes in biomass production and the accumulation of proteins, lipids, carbohydrates, and pigments [72,73]. Vitamin B12 represents one of the clearest mechanistic examples of such complementarity because bacterial cobalamin production can support B12-dependent algae under defined experimental conditions [29].

Iron acquisition provides another well-established form of cross-kingdom interaction. In some phytoplanktonic systems, bacterial siderophore production and photochemical transformation of siderophore-bound iron can alter iron availability to algal partners [74]. These interactions demonstrate that the microbial modification of micronutrient chemistry can directly influence photosynthetic organisms.

Such examples provide useful mechanistic precedents for macroalgal microbiome research. They show that metabolite exchange between algae and bacteria is biologically realistic and experimentally demonstrable. They should not, however, be used as direct evidence that equivalent exchanges occur in every macroalgal holobiont.

The important general principle is therefore metabolic complementarity, rather than universal dependence on particular microbial partners.

### 7.2. From the Classical Phycosphere to Structured Macroalgal Surfaces

The term phycosphere is most strongly associated with the chemically enriched diffusive region surrounding phytoplankton cells, where released organic compounds influence microbial chemotaxis, growth, and interaction. This conceptual framework has been extremely productive for understanding microscale algal–bacterial relationships in planktonic systems [73,75].

Macroalgal surfaces share several features with the classical phycosphere, including release of dissolved organic compounds, local nutrient gradients, microbial recruitment, and chemically mediated interactions. However, the spatial structure is fundamentally different [1,17]. A macroalgal thallus provides a persistent solid substrate on which microorganisms can attach, form biofilms, interact with surface polymers, and experience gradients associated with tissue age, light exposure, nutrient diffusion, and host physiology. Different regions of the same thallus may consequently support different microbial assemblages and functional states.

The term phycosphere can therefore be useful for describing the chemically influenced zone associated with macroalgal surfaces, but it should not obscure the distinction between a diffusive boundary environment and the attached epibiotic biofilm itself. This distinction becomes especially important when comparing planktonic and macroalgal systems. Processes such as chemotaxis toward exudates may generalize readily between them, whereas long-term biofilm development, tissue-specific colonization, and degradation of complex structural glycans are much more characteristic of multicellular macroalgal hosts.

### 7.3. Functional Redundancy and Taxonomic Variability

Across algal microbiomes, taxonomic composition is often more variable than broad functional potential. Different microbial lineages may perform overlapping ecological roles, including vitamin synthesis, nutrient transformation, carbohydrate utilization, or production of signaling molecules [4,64]. This observation is important because microbiome stability need not require taxonomic stability. A host may retain a particular ecological function even when the organisms performing it differ among locations or environmental conditions [4,64].

Evidence for such functional redundancy is especially relevant to macroalgal systems, where host-associated bacterial communities can vary geographically, seasonally, and among tissues. Genome-resolved studies further show that even closely related microbial populations may possess substantially different accessory genomes and therefore differ in functional capability.

Two complementary processes must therefore be distinguished.

The first is “taxonomic replacement with functional conservation”, in which different microorganisms provide similar ecological capabilities.

The second is “taxonomic conservation with functional divergence”, in which closely related populations differ substantially in accessory genes or metabolic pathways.

Both processes limit the ecological interpretation of taxonomic surveys alone.

This principle also complicates the concept of a “core microbiome.” A taxonomic core may consist of repeatedly detected microbial lineages, whereas a functional core may consist of metabolic capabilities maintained by different organisms. These two forms of core structure need not coincide.

Genome-resolved and functional approaches are therefore essential for determining whether ecological stability is maintained through persistent microbial populations or through redundancy among changing communities.

### 7.4. Microalgal–Bacterial Consortia and Biotechnology

Microalgal–bacterial consortia have been investigated for wastewater treatment, nutrient recovery, biomass production, biofertilizer development, carbon capture, and metabolite production [72,73,76].

In these systems, bacteria can influence algal growth through vitamin production, nutrient remineralization, removal of inhibitory compounds, changes in CO_2_ availability, and aggregation or bioflocculation, while algae provide oxygen and photosynthetically derived carbon that supports bacterial metabolism [72,73,76]. Some of these interactions have been demonstrated experimentally and, in selected cases, incorporated into pilot-scale processes.

The comparison with macroalgal biotechnology is informative but should remain limited. Microalgae can often be maintained in relatively homogeneous liquid cultures, making microbial consortia easier to manipulate and integrate into bioreactors. Macroalgae, by contrast, are multicellular hosts with spatially differentiated tissues and, particularly in open-water cultivation, continual microbial immigration from the surrounding environment.

Microbiome-engineering strategies developed for microalgal cultures therefore cannot be transferred directly to macroalgal mariculture. They nevertheless provide useful principles concerning functional complementarity, community stability, nutrient exchange, and the design of defined microbial consortia.

### 7.5. Cyanobacterial and Extremophilic Systems as Discovery Models

Cyanobacteria and extremophilic microalgae provide useful comparative models because ecological specialization can help guide the search for unusual enzymes and metabolites.

Thermophilic algae and cyanobacteria inhabiting high-temperature environments possess cellular systems adapted to conditions that destabilize many mesophilic proteins [77]. Similar reasoning can be applied to macroalgal microbiomes: microorganisms associated with hosts exposed to strong irradiance, salinity fluctuations, desiccation, hydrodynamic stress, or chemically unusual substrates may contain biochemical traits worthy of targeted investigation.

Environmental origin, however, does not demonstrate industrial suitability. Stress tolerance in situ does not necessarily translate into catalytic efficiency, stability, yield, or scalability under process conditions. Ecology can therefore guide candidate selection, but biochemical validation remains essential.

Cyanobacteria also provide well-developed examples of genome-informed natural-product discovery, including pathways for peptides, polyketides, terpenoids, and other specialized metabolites [78,79]. Their relevance to macroalgal microbiomes is primarily methodological: they demonstrate the potential of genome mining without establishing equivalent chemical richness or translational maturity in seaweed-associated communities.

### 7.6. Metagenomics Beyond Macroalgal Surfaces

Metagenomic and genome-resolved approaches are now applied across cyanobacterial, microalgal, freshwater, and marine systems, revealing previously unresolved microbial diversity and biosynthetic potential [79].

The wider marine metagenomic literature demonstrates the scale at which candidate enzymes, biosynthetic pathways, antimicrobial peptides, and other functional elements can be identified without prior cultivation. These studies provide an important methodological benchmark for macroalgal microbiome research.

Scale, however, does not remove the need for validation. Large metagenomic catalogs remain collections of predicted sequences until candidate functions are tested experimentally.

The particular strength of macroalgal microbiome metagenomics lies in combining genomic discovery with ecological context. A sequence recovered from a host-associated population gains additional biological meaning when its taxonomic origin, host, tissue location, persistence, and environmental setting are known. This integration of genomic scale with ecological specificity may therefore be one of the main advantages of macroalgal microbiomes as systems for functional discovery and bioprospecting.

### 7.7. What the Macroalgal Literature Currently Resolves Better

Comparison with broader algal microbiome research reveals several areas in which macroalgal systems provide unusually strong mechanistic or genomic resolution. The *Ulva*–bacteria morphogenesis models provide direct experimental evidence for chemically mediated developmental control and include one of the best-characterized algal bacterial morphogens, thallusin.

Genome-resolved analysis of kelp microbiomes has linked transport systems, vitamin biosynthesis, phototrophy, nutrient transformations, and glycan-utilization potential to reconstructed microbial populations rather than anonymous community sequences. Macroalgal-associated *Bacteroidota* additionally provide well-developed models for studying CAZymes and PULs involved in utilization of lineage-specific marine glycans.

These systems make macroalgal microbiomes particularly useful for connecting host biology with microbial genomic function. At the same time, mechanistic understanding remains concentrated in relatively few model hosts, notably *Ulva*, *Nereocystis*, *Ectocarpus*, and a limited number of other green, brown, and red algae. This concentration creates a major generalization problem. *Ulva’s* functional mechanisms do not automatically apply to kelps or red algae. Likewise, metabolic pathways identified in one kelp-associated microbial community may not represent universal features of Phaeophyceae-associated microbiomes.

The apparent sophistication of the field therefore coexists with substantial phylogenetic and geographical sampling bias.

### 7.8. Evidence Gaps Across the Wider Algal Microbiome Field

Several recurring limitations emerge when macroalgal and non-macroalgal systems are considered together.

First, many studies remain predominantly descriptive. Taxonomic associations are reported more frequently than experimentally validated mechanisms. Second, genomic potential is commonly interpreted more strongly than expression or metabolite data justify. Third, host–microbe relationships are often studied under one environmental condition, despite evidence that microbial function can change substantially with nutrient availability, temperature, salinity, and host physiological state. Fourth, bacteria dominate the available literature. Fungi, archaea, viruses, and microbial eukaryotes receive far less mechanistic attention despite their presence in many algal-associated communities. Fifth, comparatively few studies integrate microbial function with host fitness over ecologically meaningful timescales [5,7].

Finally, translational studies often move rapidly from gene or metabolite discovery to proposed applications without intermediate assessment of reproducibility, scalability, stability, and environmental performance. These gaps are common across the algal microbiome literature and help define the priorities required for the field to progress from association toward mechanism and from discovery toward application [53,59,66] (Table 5).

### 7.9. From Shared Principles to Ecological Divergence

Across algal microbiomes, several principles recur: photosynthetic hosts generate chemically enriched microbial habitats; microorganisms can modify nutrient and metabolite availability; taxonomic composition may vary while functional capabilities persist; and environmental conditions strongly influence the resulting interactions.

Macroalgal systems add an additional level of complexity because the host provides a persistent, spatially structured substrate on which microbial communities can differentiate over space and time. This makes macroalgae particularly suitable for studying how host lifestyle, surface chemistry, environmental exposure, and microbial recruitment interact to generate distinct functional communities.

That question becomes especially important when macroalgae enter novel ecological contexts.

Invasive species and bloom-forming algae experience rapid geographic expansion, altered microbial source pools, new environmental conditions, and intense selection for establishment and persistence. Their microbiomes therefore provide a natural test of whether successful algal hosts depend on conserved microbial partners, recruit functionally equivalent local organisms, or develop distinct microbiome configurations associated with particular ecological strategies.

The following section examines these possibilities in invasive and bloom-forming macroalgae, where the distinction between taxonomic composition, functional redundancy, dominant microbial populations, and host ecological strategy becomes particularly informative.

## 8. Microbiomes in Invasive and Bloom-Forming Macroalgae

Invasive and bloom-forming macroalgae provide a particularly informative context in which to examine the ecological significance of host-associated microbiomes. Following introduction into a new region, macroalgae encounter different microbial source pools, environmental conditions, competitors, and disturbance regimes. Bloom-forming species experience additional pressures associated with rapid biomass accumulation, drifting or rafting, tissue senescence, and large-scale changes in nutrient availability. These systems therefore raise a fundamental question: does ecological success depend on maintaining particular microbial partners, recruiting functionally equivalent local microorganisms, or assembling new microbiome configurations suited to the invaded environment [80]?

Current evidence suggests that microbiome composition can differ between native and non-native populations and can change substantially during bloom development and decline. However, comparatively few studies have demonstrated that these microbial changes directly increase invasion success or bloom persistence. Most available evidence remains taxonomic, comparative, or based on predicted functional capacity rather than experimentally demonstrated microbial causality [81,82,83].

### 8.1. Host Flexibility and the Generalist-Holobiont Hypothesis

Dependence on highly specific microbial partners could potentially constrain a host introduced into a new environment if those organisms are absent from the receiving microbial pool. Conversely, hosts capable of recruiting taxonomically different microorganisms that provide similar functions may be more resilient to geographic translocation [80,81].

This hypothesis has been examined experimentally in the invasive red macroalga *Agarophyton vermiculophyllum*. Bonthond et al. [81] compared native and non-native populations after disturbing their associated microbiota with antibiotics and exposing them to a common microbial source. Microbial communities changed substantially following disturbance, but non-native populations converged more strongly toward similar community configurations than native populations.

These results are consistent with increased flexibility in microbial recruitment by non-native populations. They do not establish that a specific microbial taxon or function causes invasiveness, but they suggest that successful introduced populations may be less dependent on retaining the precise taxonomic composition of their native microbiome [81].

This provides an important contrast with highly specific developmental interactions such as the *Ulva*–bacteria systems discussed in Section 2. Microbial dependence and microbiome flexibility need not be contradictory properties. A host may require particular functions without requiring the same microbial taxa to provide them.

Invasion success may therefore depend partly on functional replaceability: the capacity to recruit locally available microorganisms capable of restoring ecologically important functions after translocation or disturbance [81].

### 8.2. Comparative Microbiomes of Introduced Hosts

Comparative studies frequently identify differences between the microbiomes of introduced hosts and those of native species or populations, but the ecological interpretation of these differences remains challenging [80,84].

A useful related example is the invasive seagrass *Halophila stipulacea*. Aires et al. [84] reported differences in bacterial community composition between invasive *H. stipulacea* and native Caribbean seagrasses, including differences in taxa associated elsewhere with stress tolerance, nutrient acquisition, microbial interference, and plant-growth-promoting functions.

Predicted functional profiles also differed among hosts. However, because much of the functional interpretation was derived from amplicon-based taxonomic data, these predictions should be regarded as hypotheses regarding potential community functions rather than direct measurements of metabolic activity [84].

The comparison nevertheless highlights a broader methodological issue relevant to invasive macroalgae: taxonomic differences alone cannot reveal whether two microbiomes are functionally different, while predicted whole-community functions cannot determine which organisms carry those functions.

Genome-resolved comparisons of native and non-native macroalgal populations remain relatively scarce. Such analyses could determine whether invasion is associated with taxonomic replacement while maintaining functional redundancy, acquisition of new metabolic capacities, or disproportionate expansion of particular microbial populations [80].

### 8.3. Bloom-Forming Macroalgae and Microbial Succession

Large-scale macroalgal blooms provide a related but mechanistically distinct ecological context [82,83].

Studies of recurring golden tides caused by *Sargassum horneri* and green tides caused by *Ulva prolifera* in the Yellow Sea show that bloom-forming algae harbor bacterial communities distinct from surrounding seawater and that community composition changes with host condition [82,83].

In *S. horneri*, microbial communities differ between healthy and declining thalli, while functional predictions implicate processes related to nutrient transport and utilization. These observations are compatible with microbial participation in the changing nutritional environment of drifting macroalgae but do not demonstrate microbial nutrient provisioning to the host [82].

Comparative analysis of *S. horneri* and *U. prolifera* has additionally identified different dominant microbial groups. Gammaproteobacteria and Bacteroidia are prominent within some *Sargassum* sp.-associated communities, including genera such as *Vibrio* and *Marinomonas* [83]. Because some strains within these genera possess algicidal activity, their occurrence raises hypotheses about possible microbial contributions to bloom decline [83].

However, genus-level detection cannot establish algicidal activity by the populations present in a particular bloom. Strain-resolved genomic or experimental evidence is required before such ecological functions can be assigned [83].

The most likely conclusion is therefore that bloom-associated microbiomes are dynamic and respond to host and environmental states. Whether these microbial changes actively promote bloom expansion or collapse, or instead respond to changing host physiology, remains incompletely resolved [82,83].

### 8.4. From Invasion-Associated Microbiomes to Functional Attribution

The invasion and bloom literature exposes a central problem in comparative microbiome ecology. A whole-community functional signal can arise because a function is broadly distributed across many taxa, concentrated within a specialized guild, or disproportionately contributed by one dominant microbial population. These scenarios have very different biological meanings. A functional difference distributed across many taxa may indicate community-wide ecological restructuring. A function restricted to a small guild points toward specialization. A signal dominated by one population may instead indicate that the key ecological event is the recruitment or proliferation of that particular microorganism.

Importantly, dominance should not automatically be treated as statistical contamination. If a host repeatedly supports a microbial population that becomes numerically dominant, that association may itself represent an ecologically meaningful feature of the holobiont. Comparative metagenomics should therefore combine intact-community analysis with explicit taxonomic attribution rather than relying solely on aggregated pathway abundance.

This distinction provides the basis for the following case study, which examines functional differences between *Rugulopteryx okamurae* and *Sargassum* sp. holobionts in the Azores and asks whether apparent pathway-level contrasts reflect broad community differences or the contribution of individual dominant taxa.

## 9. An Illustrative Taxon-Attribution Case Study of *Rugulopteryx okamurae* and *Sargassum* sp. Holobionts in the Azores

### 9.1. Study Context and Analytical Rationale

A shotgun-metagenomic comparison of the holobionts associated with *Rugulopteryx okamurae* and *Sargassum* sp. from Azorean waters was presented at MICROBIOTECH 2025 Conference, in Ponta Delgada [85]. The two hosts represent contrasting ecological settings: *R. okamurae* is a benthic, substrate-attached brown macroalga capable of forming dense and persistent stands, whereas *Sargassum* sp. can occur in floating or drifting rafts, exposing its surface-associated microbiota to a more mobile and continuously changing environment [86,87,88].

The original analysis used the SqueezeMeta pipeline [89] to predict open reading frames (ORFs), assign taxonomy, annotate KEGG Orthology identifiers, and generate pathway-associated TPM (transcripts per million) values. Community-level aggregation identified several apparent functional contrasts between the two holobionts. However, because pathway-level TPM integrates the contribution of every annotated ORF regardless of taxonomic origin, highly abundant microbial populations can disproportionately influence the apparent functional profile of an entire community.

Reanalysis of the ORF-level dataset therefore addressed three complementary questions: what functional potential characterizes each intact holobiont; which microbial populations contribute disproportionately to those functional differences; and which contrasts remain after the contribution of a dominant population is considered separately. This analysis identified a pronounced asymmetry involving the genus *Cobetia*, which contributed a large fraction of the *Rugulopteryx* metagenomic signal but was nearly absent from *Sargassum* sp. The resulting taxon-attribution analysis therefore provides an opportunity to distinguish intact-community functional profiles from pathway differences that are not reversed by dominant-taxon exclusion.

Because the present comparison is based on the available metagenomic datasets rather than a replicated population survey, the profiles described below should be interpreted as dataset-specific and hypothesis-generating rather than species-wide characteristics (Figure 2).

#### Methodological Considerations in Macroalgal Microbiome Sampling and Sequencing

Comparisons across macroalgal microbiome studies, including those synthesized in this review, are complicated by several methodological sources of variation that are not always reported or standardized.

Thallus sampling and washing. Because macroalgal surfaces host a mixture of tightly attached and loosely associated microorganisms, the sampling protocol used—swabbing, brief rinsing, or more extensive washing and sonication protocols intended to remove all loosely attached cells—directly affects which fraction of the community is recovered. A widely used protocol for selective extraction of surface-associated bacterial DNA from macroalgae was described by Burke et al. [43] and has been adapted with variations (e.g., combined washing and ultrasonication steps) across subsequent studies; differences in wash stringency between studies can therefore contribute to apparent differences in community composition that do not reflect true biological variation.

Host-DNA depletion. Macroalgal tissue DNA can dominate shotgun sequencing libraries prepared from whole-thallus material, and different host-DNA depletion approaches recover different proportions and compositions of the associated microbial community. Comparative evaluations of host-depletion kits in other host-associated marine systems (e.g., seagrass) have shown that depletion method choice measurably affects which microbial taxa, including previously undetected members, are recovered in the resulting metagenome [90]. The present case study did not apply a dedicated host-DNA depletion step prior to sequencing, a limitation now noted explicitly in Section 9.12.

Absolute versus relative abundance. Most macroalgal microbiome studies, including the amplicon- and metagenome-based literature reviewed throughout this manuscript, report relative abundance measures (e.g., percent of reads, TPM) rather than absolute cell or organism counts. Because relative abundance is intrinsically compositional, an increase in one taxon’s reported abundance can reflect either a true increase in that taxon or a decrease in others, a distinction relevant to the *Cobetia* dominance findings discussed in Section 9 and now addressed explicitly in Section 9.12 [91].

Contamination controls, batch effects, and reference-database coverage. Cross-study comparison is further complicated by inconsistent use of negative extraction/sequencing controls, by batch effects introduced when samples are processed or sequenced in different runs or facilities, and by the incompleteness of current reference databases for marine bacterial taxa, which can leave a substantial fraction of reads unclassified or assigned only to broad taxonomic or functional categories (see also the unclassified-fraction finding reported for our own dataset in Section 9.2). None of the literature synthesized in this review can be assumed to have addressed all of these sources of variation uniformly, and cross-study comparisons should be interpreted with this heterogeneity in mind [6,45].

### 9.2. Taxonomic Contrasts Between the Two Holobionts

At the class level, Gammaproteobacteria dominate both microbiomes, accounting for approximately 63.9% of total community TPM in *Rugulopteryx* and 71.5% in *Sargassum sp*. Bacteroidia and Alphaproteobacteria are comparatively more represented in *Rugulopteryx*, whereas Fusobacteriia are more prominent in *Sargassum* sp. *Rugulopteryx* also contains a wider range of low-abundance bacterial classes, although their individual contributions to total TPM remain small. A substantial fraction of total TPM could not be assigned to a named taxonomic class in either holobiont (14.5% in Rugulopteryx; 25.7% in Sargassum sp.), and this uncertainty in taxonomic assignment should be borne in mind when interpreting the apparent dominance of individual bacterial groups.

The broad Gammaproteobacterial similarity conceals a major difference in community architecture. Within *Rugulopteryx*, the genus *Cobetia* accounts for approximately 66.8% of the Gammaproteobacterial TPM and 42.7% of total community TPM. In *Sargassum* sp., *Cobetia* represents only approximately 0.1% of the Gammaproteobacterial fraction and 0.07% of total community TPM.

This pronounced difference was cross-checked using an alternative abundance metric, mapped-read counts: approximately 48.9% of mapped reads in the *Rugulopteryx* dataset were attributed to *Cobetia*, compared with approximately 0.05% in *Sargassum* sp. The agreement between TPM-weighted and read-based estimates indicates that the observed *Cobetia* dominance is not simply an artifact of KEGG pathway aggregation. Inspection of the contig-level distribution further indicated that the signal was not attributable to a single anomalous contig.

The two communities therefore differ not merely in the relative proportions of broad bacterial classes, but in the way those classes are internally structured. The *Rugulopteryx* microbiome is strongly influenced by one dominant *Halomonadaceae* genus, whereas the Gammaproteobacterial component of *Sargassum* sp. is distributed across a greater number of genera, including *Psychromonas*, *Vibrio*, *Marinomonas*, and others.

Whether the exceptional abundance of *Cobetia* represents a stable feature of the *R. okamurae* microbiome across individuals, sites, seasons, and invaded populations remains to be established. The exclusion analysis represents a taxonomic sensitivity test rather than removal of an artefactual component of the microbiome. *Cobetia* remains part of the intact *Rugulopteryx* holobiont; the analysis determines which functional contrasts persist independently of its unusually large genomic contribution. The methodological workflow used for the taxon-attribution reanalysis is summarized in Box 1.

Box 1 **Methodological workflow for the taxon-attribution reanalysis.**

**Data source.** Shotgun metagenomic sequence data for the *Rugulopteryx okamurae* and *Sargassum* sp. holobionts were generated as part of the study presented at MICROBIOTECH 2025 Conference [85] and have since been deposited in the NCBI Sequence Read Archive under BioProject accession **PRJNA1521022**. Raw paired-end sequencing reads are available under BioSample accessions **SAMN62786275** (*Rugu-Meta-N2033*, *Rugulopteryx okamurae*) and **SAMN62786276** (*Sarg-Meta-N2034*, *Sargassum* sp.), corresponding to SRA run accessions **SRR40416375** and **SRR40416374**, respectively. Species-level identification of the *Sargassum* specimen was attempted independently of the metagenomic dataset: COI and 28S rRNA barcode regions were PCR-amplified from extracted DNA using primers provided by T. Cerqueira (OKEANOS Institute), Sanger-sequenced by a commercial provider, and the resulting sequences queried against GenBank via BLAST. The BLAST results returned multiple high-identity hits to both *Sargassum natans* and *Sargassum fluitans*, precluding unambiguous species-level assignment; the taxon is therefore reported as *Sargassum* sp. throughout this manuscript. The specimen was collected directly from the water column in a free-floating (pelagic) state, rather than as beach-stranded material, consistent with its treatment throughout this manuscript as representative of the mobile, raft-forming ecological state. This ambiguity is consistent with well-documented molecular limitations of COI and 28S barcoding within this taxonomically complex genus [92,93]. Shotgun metagenomic assembly, Prodigal ORF prediction, and DIAMOND alignment against the KEGG Orthology database were performed with the SqueezeMeta pipeline (version 1.6.5.post1), producing an ORF-level table with per-ORF taxonomic assignment, KEGG Orthology ID, KEGG pathway annotation (KEGGPATH), and per-holobiont TPM. All taxon-attribution analyses described below were performed on this ORF-level table using custom Python 3.8.19 scripts with pandas 2.0.3. [89]. Taxonomic assignments throughout this manuscript follow GTDB nomenclature, consistent with the raw taxonomic assignments produced by the SqueezeMeta pipeline (e.g., “p_Pseudomonadota,” “p_Bacteroidota,” “p_Actinomycetota” at the phylum level).

Step 1—pathway parsing. The KEGGPATH field was parsed per ORF and restricted to canonical, numbered KEGG pathway maps filed under four microbial-relevant top-level categories (Metabolism, Genetic Information Processing, Environmental Information Processing, Cellular Processes); the “Organismal Systems” and “Human Diseases” top-level categories were excluded, as these contain pathway maps centered on host/eukaryotic physiology that can accumulate shared-KO signal in a bacterial metagenome without representing real community biology. An additional 91 individual pathway maps identified as eukaryote-, plant-, animal-, or fungus-specific despite being filed under a retained category (e.g., “Protein processing in endoplasmic reticulum,” “Peroxisome,” “Hippo signaling pathway”) were also excluded; the complete list and rationale are provided in the supplementary code repository. BRITE-hierarchy protein-family classifications (e.g., “Transporters,” “Ribosome”) were excluded as before, as structural groupings rather than numbered pathway maps.

Step 2—pathway-level TPM aggregation. ORF-level TPM was summed per canonical pathway, separately for each holobiont, to obtain baseline intact-community pathway-level abundance.

Step 3—taxon-attribution (exclusion) test. Dominant taxa were identified by overall community TPM share; pathway-level TPM was recomputed after computationally excluding all ORFs assigned to a given taxon. Two comparisons are reported. First, the comparative direction (which holobiont has the higher TPM) was compared before and after exclusion using the post-exclusion TPM values as originally normalized (i.e., without adjusting for the amount of signal removed); pathways whose direction reversed under this comparison were classified as apparently *Cobetia*-sensitive. Second, and reported as the primary result throughout Section 9, post-exclusion TPM was renormalized to the total post-exclusion (residual) community TPM for each holobiont, expressing each pathway’s representation as a percentage of the community that remains after *Cobetia* exclusion rather than carrying over the intact-community normalization; direction reversal under this renormalized comparison provides a more conservative and interpretable measure of which pathway contrasts are genuinely attributable to *Cobetia*’s dominance, as opposed to an artifact of the amount of signal that happened to be removed.

Step 4—full-pathway-set core ranking. The same exclusion test was extended to all 162 canonical microbial pathways detected in the combined dataset following the Step 1 restriction, ranked by percent difference in renormalized post-exclusion TPM between holobionts (0% = perfectly balanced), with a minimum combined-TPM floor (≥200) applied to exclude near-undetected pathways from balanced-pathway claims. The identity of the resulting top-ranked balanced pathways was confirmed stable across alternative floors of 100, 300, and 500 combined TPM.

Step 5—taxonomic composition and *Cobetia*-specific breakdown. Class- and genus-level TPM-weighted composition was computed per holobiont; the dominant taxon’s (*Cobetia*) contribution to its own class and to total community TPM was computed, and cross-checked against raw mapped-read counts as an alternative abundance metric (see Section 9.2 for why this constitutes a cross-check rather than independent validation); its contig-level distribution was also examined to rule out single-contig assembly artifacts.

*Full pathway-level output tables and analysis scripts are available as Supplementary Material.*

### 9.3. Identification of the Cobetia Effect Through Pyruvate Metabolism

The disproportionate contribution of *Cobetia* was not hypothesized in advance. It emerged during validation of the pathway rankings against the original Core/Benthic-enriched/Pelagic-enriched classification.

Pyruvate metabolism provided the critical discrepancy. At the whole-community level, the pathway appeared higher in *Rugulopteryx*. Inspection of the highest-TPM individual ORFs, however, showed that many of the strongest individual contributions were higher in *Sargassum* sp. Thus, the most conspicuous individual ORFs favored *Sargassum* sp., while the aggregated pathway total favored *Rugulopteryx*.

Taxonomic reaggregation resolved this apparent contradiction. *Cobetia* alone accounted for approximately 44% of the total pyruvate-metabolism TPM in *Rugulopteryx*. Its contribution was distributed across numerous moderately represented ORFs rather than concentrated in one or a few dominant genes. Consequently, no single *Cobetia*-associated ORF appeared among the strongest individual pathway contributors, while their cumulative abundance substantially increased the total *Rugulopteryx* pathway signal. When *Cobetia*-assigned ORFs were excluded, pyruvate metabolism changed direction and became higher in *Sargassum* sp.

This observation demonstrated that community-level pathway aggregation can produce an apparently strong holobiont-level difference through the cumulative genomic contribution of one abundant population. We note that this initial observation used the carried-over (non-renormalized) comparison described in Section 9.4; once post-exclusion values are renormalized to the residual community total, pyruvate metabolism’s enrichment direction is in fact retained rather than reversed (Section 9.4). The discrepancy itself is informative: it demonstrates concretely how the choice of normalization can determine whether a pathway appears to flip, which is precisely why the renormalized comparison, rather than the carried-over one, is used as the primary result throughout the remainder of Section 9. This initial observation nonetheless motivated extending the taxonomic attribution analysis systematically across the major KEGG pathways.

### 9.4. Dominant-Taxon Sensitivity of the Top-25 KEGG Pathways

The 25 canonical microbial KEGG pathways with the greatest combined intact-community TPM initially suggested extensive functional divergence between the two holobionts. Recalculation after exclusion of *Cobetia*-assigned ORFs, with post-exclusion TPM renormalized to the total residual-community TPM, substantially changed this interpretation, though less drastically than an uncorrected comparison suggests. Five of the 25 pathways, representing 20% of the Top-25 set, changed the direction of their *Rugulopteryx*–*Sargassum* sp. contrast after *Cobetia* exclusion and renormalization. Twenty retained the same direction. (If post-exclusion values are not renormalized to the residual community—i.e., if the intact-community normalization is carried over unchanged—14 of the 25 pathways, or 56%, appear to change direction; this larger, uncorrected figure substantially overstates the sensitivity of the comparison to *Cobetia* exclusion, for the reasons discussed in Section 9.12.) Pathways whose *Rugulopteryx*–*Sargassum* sp. contrast is attributable to *Cobetia*’s genomic contribution, rather than to a broader community-level difference, included ABC transporters, quorum sensing, ribosome, homologous recombination, and biofilm formation (*Pseudomonas aeruginosa*). In contrast, glyoxylate and dicarboxylate metabolism and valine/leucine/isoleucine degradation retained their *Rugulopteryx*-associated direction; bacterial chemotaxis, flagellar assembly, porphyrin and chlorophyll metabolism, and biofilm formation (*Vibrio cholerae*) retained a stronger *Sargassum* sp.-associated signal; and purine metabolism, pyruvate metabolism, glycine/serine/threonine metabolism, pyrimidine metabolism, oxidative phosphorylation, cysteine/methionine metabolism, glycolysis/gluconeogenesis, alanine/aspartate/glutamate metabolism, propanoate metabolism, methane metabolism, carbon fixation pathways in prokaryotes, amino sugar and nucleotide sugar metabolism, aminoacyl-tRNA biosynthesis, and the two-component system all retained their original direction as well. The intact-community and *Cobetia*-excluded profiles answer different questions. The former describes the functional potential of the biological communities as recovered, including *Cobetia*. The latter identifies pathway contrasts that persist when the disproportionate contribution of this dominant genus is considered separately, once properly renormalized to the residual community (Figure 3).

### 9.5. Reassessment of the Shared Functional Backbone

The same taxon-exclusion procedure was applied to the pathway set used in the original Core/Benthic-enriched/Pelagic-enriched classification, restricted to the same canonical microbial KEGG categories used throughout Section 9, and with post-exclusion pathway TPM renormalized to the total residual-community TPM rather than carried over from the intact-community normalization (see Section 9.4). Under this corrected comparison, 5 of the 25 pathways in the original classification change enrichment direction after *Cobetia* exclusion and renormalization: nicotinate and nicotinamide metabolism, glycerophospholipid metabolism, phenylalanine/tyrosine/tryptophan biosynthesis, one-carbon pool by folate, and biofilm formation (*Pseudomonas aeruginosa*). This is a substantially smaller effect than an uncorrected comparison suggests—18 of the same 25 pathways change direction if post-exclusion values are not renormalized to the residual community—illustrating how much of the originally reported instability was attributable to retaining the intact-community normalization rather than to *Cobetia*’s influence itself. Re-examining the 12 pathways originally placed in the “Core” category under this corrected method shows that most of them are, in fact, closely balanced between the two holobionts once taxonomic contribution is properly accounted for. Ranked by relative percentage difference between the renormalized *Rugulopteryx* and *Sargassum* profiles, these 12 pathways separate into two groups at a natural break in the data: eight pathways cluster tightly between 0% and 12.0% difference (one-carbon pool by folate, 0%; phenylalanine/tyrosine/tryptophan biosynthesis, 1.0%; purine metabolism, 1.9%; nicotinate and nicotinamide metabolism, 4.6%; pantothenate and CoA biosynthesis, 5.2%; monobactam biosynthesis, 5.4%; pyrimidine metabolism, 8.7%; glycerophospholipid metabolism, 12.0%), while the remaining four are markedly more skewed, beginning at 16.7% (two-component system, 16.7%; amino sugar and nucleotide sugar metabolism, 25.1%; alanine/aspartate/glutamate metabolism, 29.7%; histidine metabolism, 40.9%). The gap separating these two groups (4.7 percentage points, between 12.0% and 16.7%) is the largest gap between any two adjacent values in the ranked set, more than three times the size of any other gap within the lower cluster; consequently, any threshold placed between 12.0% and 16.7% partitions the 12 pathways identically. We adopt 15% as a round value within this empirically defined interval to classify a pathway as “closely balanced.” Under this criterion, eight of the twelve pathways originally placed in the “Core” category are closely balanced, supporting rather than undermining the original designation for these pathways; the remaining four are better described as holobiont-associated rather than shared/core functions. Of these four, the two-component system retains a modest *Sargassum*-leaning skew, while amino sugar and nucleotide sugar metabolism, alanine/aspartate/glutamate metabolism, and histidine metabolism retain a consistent *Rugulopteryx*-leaning skew both before and after exclusion. Extending the comparison across all 162 canonical microbial pathways detected in the dataset provided a broader basis for identifying functions closely balanced between the two datasets and confirmed that this comparison is subject to the same normalization issue identified elsewhere in Section 9.

Ranking pathways by percentage difference in raw, carried-over post-exclusion TPM (i.e., without renormalizing to each holobiont’s own residual-community total)—an uncorrected method—appeared to show the citrate cycle (TCA cycle), oxidative phosphorylation, and butanoate metabolism as closely matched between holobionts. This appearance is an artifact of the same normalization issue discussed in Section 9.4: because *Rugulopteryx*’s residual community (after *Cobetia* exclusion) totals only 57.4% of its original TPM, while *Sargassum*’s residual community is essentially unchanged (99.9% of its original total), pathways with similar raw post-exclusion TPM values in fact represent substantially different shares of each holobiont’s residual community. Once properly renormalized, the citrate cycle, oxidative phosphorylation, and butanoate metabolism each show a 55–57% relative difference between holobionts—a moderate, *Rugulopteryx*-leaning skew, in this comparison. A different set of pathways emerges as closely balanced in this dataset under the corrected, renormalized comparison, restricted to pathways with combined post-exclusion TPM ≥ 200: purine metabolism (23,434 TPM, 1.9% difference), quorum sensing (23,074 TPM, 3.6%), homologous recombination (12,353 TPM, 6.4%), bacterial secretion system (10,088 TPM, 7.0%), pentose phosphate pathway (10,345 TPM, 7.9%), and pyrimidine metabolism (14,576 TPM, 8.7%) are all both high-abundance and closely balanced. Two of these (quorum sensing and homologous recombination) are also among the five pathways identified in Section 9.4 as *Cobetia*-sensitive under the Top-25 comparison—consistent with the interpretation that a pathway sitting very close to a 50/50 balance point is exactly the kind of pathway whose apparent direction is most easily tipped by a single dominant taxon’s exclusion. This ranking of balanced pathways was stable across alternative abundance floors (100, 300, and 500 combined TPM; 10/10, 10/10, and 9/10 overlap with the primary threshold respectively), so the identification of these pathways as balanced is not an artifact of the specific floor chosen—only the earlier, uncorrected identification of the citrate cycle/oxidative phosphorylation/butanoate trio was. Oxidative phosphorylation is particularly informative because it had previously been used to support the interpretation that *Rugulopteryx* hosts a more energetically demanding microbiome, and can appear as part of a balanced shared backbone when the comparison is not properly renormalized. Neither interpretation is supported once the comparison is correctly renormalized: oxidative phosphorylation shows a moderate *Rugulopteryx*-leaning skew (community share 1.20% versus 0.67%, roughly 1.8-fold), consistent with the original, pre-reanalysis observation, though substantially more modest than a simple ratio of intact-community TPM would suggest, and not distinguishable at this stage from the broader class of *Rugulopteryx*-leaning central-metabolism pathways discussed elsewhere in this section. The functional divergence between the two holobionts therefore does not resolve into a single high-abundance “shared backbone” once the comparison is properly renormalized. Central carbon and energy metabolism pathways (TCA cycle, oxidative phosphorylation, butanoate metabolism) are, on proper renormalization, modestly but consistently *Rugulopteryx*-leaning rather than balanced, while a distinct set of pathways—several involved in nucleotide metabolism, quorum sensing, homologous recombination, and secretion—are closely balanced between holobionts in this dataset. We report both findings, and the methodological correction that separates them, as the more precise and more defensible characterization of shared versus holobiont-specific function than an uncorrected or only partially corrected comparison would provide.

### 9.6. Rugulopteryx: A Resource-Recycling Functional Profile

Two metabolic pathways remain consistently higher in *Rugulopteryx* across the *Cobetia*-exclusion comparisons: glyoxylate and dicarboxylate metabolism, and valine, leucine and isoleucine degradation. Both pathways are associated principally with catabolic utilization and recycling of organic substrates rather than generalized increases in biosynthetic or respiratory capacity [94,95].

The glyoxylate cycle allows microorganisms to assimilate two-carbon compounds such as acetate while bypassing the CO_2_-releasing decarboxylation steps of the complete TCA cycle. It therefore permits conservation of carbon skeletons during growth on C2 compounds and on products derived from lipid and organic acid metabolism [94].

Branched-chain amino-acid degradation similarly allows valine, leucine and isoleucine to contribute carbon skeletons and reducing equivalents to central metabolism [95].

Their greater genomic representation is therefore compatible with a microbiome characterized by efficient utilization of heterogeneous organic substrates and recycling of carbon- and amino-acid-derived compounds.

The benthic ecology of *R. okamurae* provides a plausible context for this profile. Persistent attachment exposes the microbial community to host exudates, tissue turnover, particulate material, and benthic organic inputs over relatively long residence times [86].

The metagenomic data do not demonstrate increased pathway activity or flux, however, nor do they demonstrate that these bacterial functions contribute causally to the formation of dense *Rugulopteryx* stands. The evidence supports a more restricted conclusion: the *Rugulopteryx*-associated microbiome contains greater representation of catabolic capacities compatible with organic-substrate scavenging and resource recycling.

### 9.7. Sargassum sp.: Motility, Surface Colonization, and Redox-Associated Potential

The *Sargassum* sp. microbiome retains a broader group of comparative signals after *Cobetia* exclusion, including porphyrin and chlorophyll metabolism, the pentose phosphate pathway, biofilm formation (*Vibrio cholerae*), bacterial chemotaxis, and flagellar assembly. A second biofilm-formation map, Pseudomonas aeruginosa, is sensitive to Cobetia exclusion under the renormalized comparison and is therefore not included among the directionally retained *Sargassum*-associated signals (see Section 9.4).

The combination of bacterial chemotaxis, flagellar assembly, and the *Vibrio cholerae* biofilm-formation signal forms a particularly coherent functional pattern. Chemotaxis and flagellar systems provide microorganisms with the capacity to detect chemical gradients and move toward favorable microsites, while this biofilm-formation pathway includes functions involved in attachment, extracellular matrix formation, intercellular signaling, and persistence on surfaces [1,75]. Together, these pathways indicate greater genomic representation of a motility–encounter–attachment axis in the *Sargassum* sp.-associated community.

This pattern is compatible with colonization and recolonization of a mobile or dynamically exposed algal surface. It does not demonstrate that microbial biofilms increase raft cohesion or cause long-distance dispersal. Floating *Sargassum* sp. transport is principally determined by host buoyancy, morphology, hydrodynamics, and oceanographic forcing; the microbial data support increased surface-colonization potential rather than a microbiome-driven dispersal mechanism [87,88].

The pentose phosphate pathway contributes NADPH, ribose-5-phosphate, redox homeostasis, biosynthesis, and nucleotide production. Its greater representation in *Sargassum* sp. is therefore compatible with differences in reducing-power requirements and oxidative-stress management, but it is not a specific marker of photooxidative stress [96].

Similarly, porphyrin and chlorophyll metabolism encompasses tetrapyrrole pathways involved in hemes, cytochromes, and related cofactors. Its greater representation indicates differences in tetrapyrrole-associated metabolism rather than direct evidence of UV protection [97].

The combined *Sargassum* sp. profile is therefore best interpreted as greater representation of microbial motility, surface-colonization, and redox-associated functional potential.

### 9.8. Ecological Synthesis of the Two Holobiont Profiles

The *Cobetia*-excluded profiles are consistent with two different ecological configurations.

The *Rugulopteryx* microbiome retains a relatively narrow signature centered on carbon- and amino-acid-catabolic pathways compatible with efficient resource recycling in a persistent benthic environment. The *Sargassum* sp. microbiome retains stronger representation of bacterial motility, chemotaxis, and surface-associated functions, together with differences in redox- and tetrapyrrole-associated metabolism. These functions are compatible with microbial recruitment and persistence on a mobile and environmentally exposed host surface.

The resulting contrast may therefore be described as a resource-recycling benthic microbiome versus a motility- and colonization-oriented mobile-surface microbiome. These profiles should not be interpreted as demonstrated microbial invasion mechanisms. Host ecology may select for the microbial functions observed rather than those microbial functions determining host ecological strategy (Figure 4).

### 9.9. Cobetia as a Distinct Ecological Signal

The taxon-attribution analysis identifies *Cobetia* as the major contributor to many intact-community pathway differences in *Rugulopteryx*. Its ecological significance, however, extends beyond its influence on pathway aggregation.

*Cobetia*-assigned ORFs are strongly represented among nutrient- and osmolyte-acquisition functions, including ABC and TRAP-type transporters, betaine/carnitine and glycine-betaine transport systems, ammonium-associated functions, and iron-complex transport.

The ORF profile also contains diguanylate cyclase-associated functions. Cyclic-di-GMP is a major bacterial second messenger involved in transitions between motile and sessile lifestyles and in regulation of surface attachment, extracellular matrix production, and biofilm formation [98]. This functional repertoire is compatible with a bacterium adapted to nutrient acquisition and regulation of a surface-associated lifestyle. The near-exclusive abundance of *Cobetia* in *Rugulopteryx* raises an additional ecological question: why does this host support such a large *Cobetia* population whereas *Sargassum* sp. does not?

Potential explanatory factors include differences in surface chemistry, host exudates, polysaccharide composition, osmolyte availability, attachment properties, microenvironmental conditions, and microbial competition. Determining which of these processes contributes to the association requires replicated ecological and experimental investigation [1,17].

### 9.10. A Testable Hypothesis: Cobetia and Surface Community Assembly

Several independently characterized members of the genus *Cobetia* produce extracellular compounds with potential effects on microbial community organization [99,100,101]. *Cobetia marina* DSMZ 4741 produces a K-antigen-like exopolysaccharide [99]. Other isolates produce glycolipid biosurfactants with antimicrobial and anti-biofilm activity [100], while *Cobetia* sp. MM1IDA2H-1 produces a biosurfactant capable of interfering with quorum sensing in a fish pathogen through signal hijacking [101]. These phenotypes demonstrate metabolic capabilities within the genus but do not establish equivalent activity in the *Rugulopteryx*-associated population.

Nevertheless, the combination of high numerical dominance, strong representation of nutrient- and osmolyte-acquisition systems, surface-lifestyle regulatory genes, and extracellular-product phenotypes documented in other *Cobetia* strains provides a testable hypothesis that the population may influence microbial community assembly at the *Rugulopteryx* surface. Such effects could involve extracellular polysaccharides, biosurfactants, quorum interference, competition for attachment sites, or other mechanisms affecting neighboring microorganisms.

Testing this hypothesis will require isolation or genome-resolved reconstruction of the relevant *Cobetia* population, followed by direct characterization of exopolysaccharide production, biosurfactant synthesis, biofilm formation, quorum interference, antimicrobial activity, host-surface adhesion, and interactions with representative members of the surrounding microbial community.

### 9.11. From Taxon Attribution to Targeted Bioprospecting

The taxon-attributed analysis also changes the bioprospecting implications of the original community-level comparison.

The persistent *Rugulopteryx*-associated signals in glyoxylate/dicarboxylate metabolism and branched-chain amino-acid degradation identify community-level capacities related to utilization of organic acids and amino-acid-derived substrates. Their technological relevance remains exploratory and requires identification and biochemical characterization of individual enzymes or metabolic pathways.

*Cobetia* provides a more specific bioprospecting target. Members of this genus are documented producers of exopolysaccharides and glycolipid biosurfactants with antimicrobial, antibiofilm, or quorum-interference activities [99,100,101]. The exceptional representation of *Cobetia* in the *Rugulopteryx* dataset therefore provides an ecological rationale for targeted isolation and characterization of the associated population.

For *Sargassum* sp., the stronger representation of bacterial chemotaxis, flagellar assembly, biofilm formation (*Vibrio cholerae*), tetrapyrrole metabolism, and the pentose phosphate pathway identifies microbial populations involved in colonization and redox-associated metabolism as priorities for genome-resolved investigation.

The appropriate progression from ecological observation to biotechnology is therefore:

Ecological association → taxonomic attribution → genome reconstruction → candidate pathway or metabolite identification → cultivation or heterologous expression → biochemical validation → technological assessment.

### 9.12. Limitations and Validation Priorities

The present case study remains based on preliminary metagenomic data and should be interpreted accordingly. The underlying shotgun metagenomic datasets were originally presented at MICROBIOTECH 2025 [85], while the raw sequence data are now publicly archived in the NCBI Sequence Read Archive under BioProject PRJNA1521022. The taxon-attribution analysis presented here remains a reanalysis of those preliminary datasets rather than a peer-reviewed genome-resolved study. Functional comparisons are based primarily on ORF-level taxonomic and KEGG attribution rather than complete MAG reconstruction. TPM values derive from DNA-based metagenomic read recruitment and therefore represent normalized genomic sequence abundance rather than transcriptional activity. TPM normalization also does not correct for genome size or gene copy number: a taxon with a larger genome, or a gene present in multiple copies, can contribute disproportionately to TPM without a corresponding difference in cell numbers or organismal abundance. Pathway-level TPM should therefore be interpreted as an estimate of normalized genomic sequence representation, not as a direct measure of organismal abundance, gene dosage, or metabolic activity. Post-exclusion pathway TPM is renormalized to the total residual-community TPM throughout this case study (Section 9.4, Box 1, Step 3), so that reported changes reflect each pathway’s representation within the community that remains after Cobetia exclusion rather than merely the amount of signal removed; the non-renormalized (carried-over-normalization) comparison is also reported alongside this result for methodological transparency, since the two approaches can disagree substantially (Section 9.4). Pathways described as more highly represented cannot be assumed to be more actively expressed in situ.

The taxon-exclusion procedure applied throughout this case study is a sensitivity analysis: it estimates how much of an observed community-level pathway contrast is attributable to the genomic contribution of a single dominant population. It is not a substitute for biological replication, strain- or MAG-resolved validation, or formal statistical testing, and its results should not be interpreted as such. The available analysis does not yet establish sufficient biological replication to support population-wide differential-abundance inference for *R. okamurae* and *Sargassum* sp. Replicated sampling across individuals, locations, seasons, and ecological conditions will therefore be necessary to determine whether the observed *Cobetia* dominance and pathway patterns are reproducible.

KEGG pathway totals also contain overlapping information because individual KOs can contribute to multiple pathways: 38.4% of pathway-annotated ORFs in this dataset are assigned to more than one retained pathway. Pathway-associated TPM values should therefore not be treated as independent metabolic measurements.

In addition, exclusion of *Cobetia* addresses one dominant population only. A pathway that is directionally retained after Cobetia exclusion may still be concentrated within another restricted functional guild rather than distributed evenly throughout the community.

Several subsequent analyses would substantially strengthen the interpretation. Genome-resolved assembly should determine whether the *Cobetia* signal derives from one dominant strain or several closely related populations. Replicated statistical comparisons should test pathway differences formally. Metatranscriptomics, metaproteomics, and metabolomics can determine whether genomic differences correspond to functional deployment, while stable-isotope approaches may resolve specific carbon- or nitrogen-processing hypotheses. Finally, cultivation and synthetic-community experiments are required to determine whether candidate microbial populations influence host physiology or microbial community assembly.

### 9.13. A Taxon-Attributed Framework for Functional Holobiont Metagenomics

The *Rugulopteryx–Sargassum* sp. comparison illustrates the importance of separating three complementary levels of functional interpretation.

Level 1—Intact-community functional profile. This represents the total functional potential encoded by all microbial populations associated with the host. At this level, *Cobetia* is legitimately part of the *Rugulopteryx* functional profile.

Level 2—Taxon-attribution functional profile. This identifies which microbial populations contribute to particular pathway signals and determines whether apparent functional differences are broadly distributed or taxonomically concentrated.

Level 3—Distributed functional profile. This evaluates which comparative pathway differences remain after dominant-population effects are considered separately. These functions are directionally retained despite taxonomic dominance, although they cannot automatically be assumed to be uniformly distributed across the remainder of the community.

This framework changes the central question posed by comparative pathway analysis. Rather than asking only which KEGG pathways are more highly represented in one holobiont than another, taxon-attributed interpretation asks which organisms encode those pathways, how much of the signal is generated by dominant populations, how broadly the functions are distributed, whether they are expressed, and whether they affect host phenotype.

The *Rugulopteryx–Sargassum* sp. comparison demonstrates why these distinctions matter. A minority of apparent pathway differences—five of the twenty-five most abundant pathways, once post-exclusion values were properly renormalized to the residual community—changed direction after the contribution of *Cobetia* was considered explicitly, while the majority retained their direction in the taxon-exclusion sensitivity analysis. At the same time, the exceptional abundance and functional repertoire of *Cobetia* emerged as one of the most biologically interesting features of the dataset.

Functional differences among macroalgal holobionts therefore cannot be interpreted adequately from community-level pathway abundance alone. Linking those functions to the microbial populations that generate them provides a more rigorous basis for connecting metagenomic variation with host ecology and for identifying specific microorganisms and pathways for subsequent experimental and biotechnological investigation.

## 10. Conclusions and Outlook

Macroalgal microbiome research has moved rapidly from taxonomic description toward increasingly detailed reconstruction of microbial functional potential. This shift has revealed that seaweed-associated microorganisms can participate in host development, nutrient transformation, environmental sensing, carbohydrate utilization, and secondary metabolism. However, the strength of evidence supporting these functions remains highly uneven.

A central distinction throughout this review is that genomic potential is not equivalent to ecological activity. Detection of pathways for cobalamin biosynthesis, nitrogen transformation, DMSP metabolism, carbohydrate degradation, or specialized-metabolite production establishes biochemical capacity, but not necessarily expression, metabolite transfer, host benefit, or causality. Mechanistic understanding therefore requires progression from genomic prediction toward functional expression, biochemical activity, metabolite exchange, host response, and experimental validation.

Taxonomic composition and functional capacity are likewise not interchangeable. Macroalgal microbiomes can vary substantially among hosts, tissues, locations, seasons, and environmental conditions while retaining overlapping ecological functions through functional redundancy. Conversely, closely related microbial populations can differ markedly in accessory genes and metabolic capabilities. Genome-resolved and taxon-resolved approaches are therefore essential for determining not only which functions are present, but which organisms encode them and how broadly those functions are distributed across the community.

This becomes particularly important when microbiomes contain dominant microbial populations. Whole-community pathway abundance can reflect the disproportionate genomic contribution of one taxon rather than a broadly distributed community property. The *Rugulopteryx okamurae–Sargassum* sp. comparison examined here illustrates this problem. Explicit taxonomic attribution substantially altered the interpretation of several KEGG pathway contrasts and showed that intact-community and dominant-taxon-sensitive analyses answer different biological questions. The significance of *Cobetia* lies not only in its influence on pathway aggregation, but also in its emergence as a specific ecological and biotechnological target for future genome reconstruction, cultivation, and functional testing.

These principles have direct consequences for bioprospecting. Macroalgal microbiomes contain substantial enzymatic and biosynthetic diversity, particularly in carbohydrate-active enzymes, polysaccharide-utilization systems, extracellular products, and specialized-metabolite pathways. Yet sequence discovery alone does not establish technological relevance. Candidate genes and biosynthetic clusters must be linked to producing organisms, expressed, characterized biochemically, and evaluated for stability, yield, substrate specificity, process compatibility, safety, and scalability.

Ecological context can nevertheless improve discovery efficiency. Host polysaccharide chemistry can guide searches for specialized glycan-degrading enzymes, persistent surface-associated populations can be prioritized for adhesion or extracellular-polymer traits, and microorganisms exposed to strong environmental fluctuations may provide candidates for stress-tolerant biochemical systems. In this sense, ecology provides the rationale for candidate selection, metagenomics provides the discovery framework, and experimental validation determines whether the predicted function has practical value.

Future progress will depend on stronger biological replication across hosts, tissues, seasons, and locations; more extensive genome reconstruction; and tighter integration of metagenomics with metatranscriptomics, metaproteomics, metabolomics, isotope tracing, cultivation, and experimental community manipulation. Spatially resolved approaches will also be important for capturing heterogeneity across macroalgal surfaces and tissues. At the same time, fungi, archaea, viruses, and other microbial eukaryotes require far greater attention if the macroalgal holobiont is to be understood beyond its current bacterial bias.

This narrative review, in common with much of the current literature, draws disproportionately on a small number of intensively studied model systems—predominantly Ulva, Nereocystis luetkeana, and Ectocarpus—for its mechanistic and genome-resolved evidence. This concentration reflects the state of the field rather than a deliberate scope choice: these systems have accumulated experimental tractability (axenic culture methods, genetic tools, or long-running genome-resolved sampling programs) that most other macroalgal genera currently lack. Conclusions framed as general properties of “macroalgal microbiomes” should therefore be read with this concentration in mind, and extension to brown, red, and green macroalgal lineages beyond these model systems should be treated as a hypothesis rather than an established generalization [5].

Host-associated bacterial community structure does not always track geographic distance in the way planktonic marine microbial communities do. In a continental-scale study of the kelp Ecklonia radiata spanning a 10° latitude and 35° longitude across Australia, variation in host-associated bacterial communities was better explained by host condition than by geography [102]. A separate study of related kelp systems similarly found that host genetics and phenotype, rather than geographic distance alone, structured the microbiome [103]. These findings caution against assuming that geographically distant populations of the same macroalgal species will show proportionally distant microbiomes, and this review has not systematically addressed biogeographic variation in the taxa it discusses.

Whether macroalgal-associated bacteria are transmitted vertically (parent to offspring, or across life-history stages) or acquired horizontally (from the surrounding seawater at each generation) remains poorly resolved for most systems discussed in this review. A study of kelp gametophyte microbiota found that gametophyte and parental sporophyte communities resembled each other more than either resembled the water column, consistent with at least partial transmission across life stages [23], but comparable direct tests are lacking for most of the macroalgal systems discussed elsewhere in this review, including Rugulopteryx and Sargassum.

Longitudinal sampling of macroalgal microbiomes across multiple timepoints, and comparative sampling of the surrounding seawater and sediment alongside the host, remain uncommon in the literature this review draws on. Where such comparisons exist, they generally show host-associated communities to be compositionally distinguishable from surrounding seawater while still sharing a subset of taxa with it [64], underscoring the need for paired environmental sampling—a design not used in Section 9 case study in this review, which lacks paired seawater or sediment samples.

Most experimental work on macroalgal holobiont responses to environmental change, including the salinity-acclimation and cold-tolerance evidence discussed elsewhere in this review, addresses single stressors in isolation. How macroalgal holobionts respond to the simultaneous or sequential combination of stressors relevant to ongoing environmental change (e.g., warming combined with nutrient enrichment, or combined with reduced salinity) is largely untested for the systems reviewed here and represents an important gap for future work.

A useful progression for future ecological studies is therefore as follows:

Community composition → genomic potential → taxonomic attribution → functional expression → metabolite exchange → host phenotype → experimental causality.

For biotechnology, a parallel sequence applies the following:

ecological association → candidate discovery → genome-resolved attribution → biochemical validation → process characterization → application testing.

The field does not primarily need longer inventories of microbial genes or predicted pathways. Its next advance will come from determining which organisms perform particular functions, under which environmental conditions, and with what consequences for the host or for technological application. Macroalgal holobionts are therefore best viewed as dynamic ecological systems in which host traits, environmental conditions, microbial recruitment, functional redundancy, and population dominance jointly shape the functional repertoire of the microbiome. Deciphering these interactions will be essential both for understanding macroalgal ecology and for translating microbial genomic diversity into reproducible and sustainable biotechnology.

## Figures and Tables

**Figure 1:**
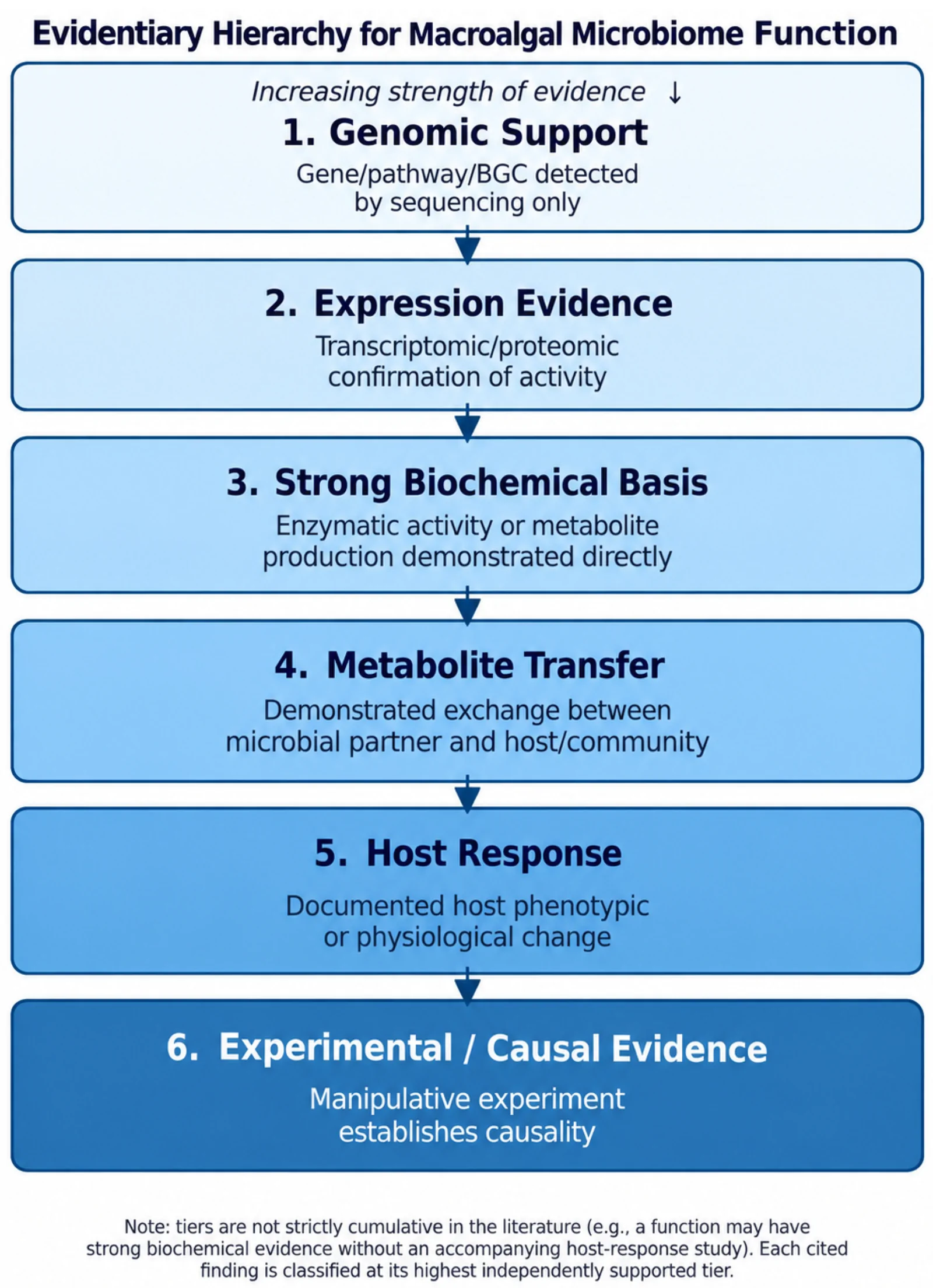
Evidentiary hierarchy used throughout this review to classify the strength of evidence supporting a proposed macroalgal microbiome function. Evidence is organized into six tiers of increasing strength: (1) genomic support, where a gene, pathway, or biosynthetic gene cluster has been detected by sequencing alone; (2) expression evidence, where transcriptomic or proteomic data confirm the gene or pathway is actively expressed; (3) strong biochemical basis, where enzymatic activity or metabolite production has been demonstrated directly, typically in vitro or via heterologous expression; (4) metabolite transfer, where exchange of a specific metabolite between the microbial partner and the host or another community member has been demonstrated; (5) host response, where a measurable phenotypic or physiological change in the host has been documented in association with the function; and (6) experimental or causal evidence, where a manipulative experiment (e.g., knockout, gnotobiotic reconstruction, or synthetic community assembly) establishes a causal role for the function in the observed outcome. Tiers are not strictly cumulative in the primary literature—a function may, for example, have strong biochemical evidence without an accompanying host-response study—and each finding discussed in this review is classified at its highest independently supported tier (see “Literature Search and Evidence Classification,” Introduction). This figure is referenced throughout the review (Section 2.4, Section 3.6, Section 4.8, Section 5.6 and Section 6.9) in place of restating the underlying argument in each section individually.

**Figure 2:**
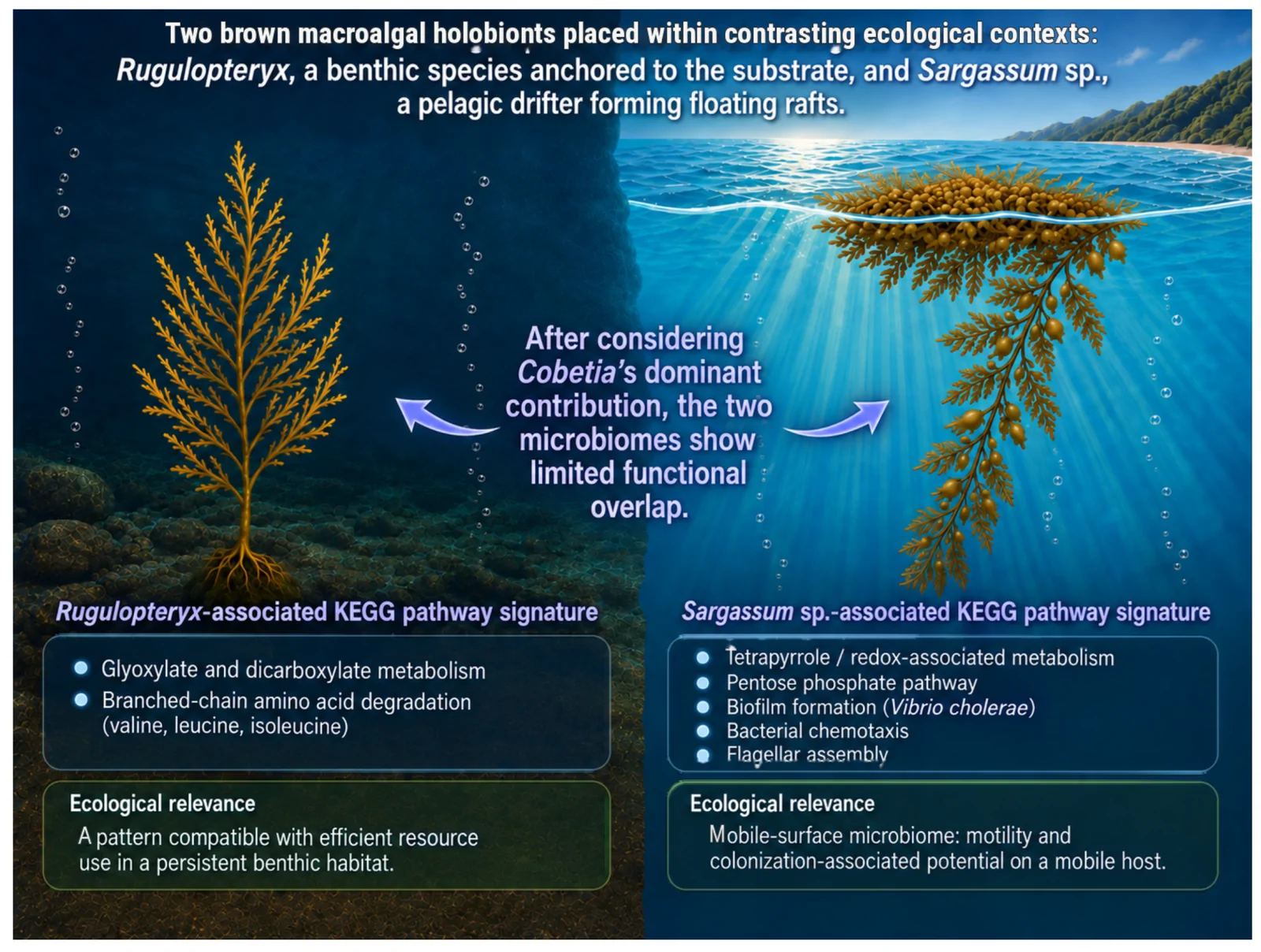
Ecological context of the two macroalgal holobionts and principal KEGG pathway-associated contrasts identified after exclusion of *Cobetia*-assigned ORFs and renormalization to the residual community (Section 9.4 and Section 9.5). Left: *Rugulopteryx okamurae*, a benthic species anchored to the substrate. Right: *Sargassum* sp., represented in a floating, raft-forming ecological context. The *Rugulopteryx*-associated microbiome retains higher pathway-associated TPM for glyoxylate and dicarboxylate metabolism and valine, leucine and isoleucine degradation. The *Sargassum* sp.-associated microbiome retains higher representation of tetrapyrrole/redox-associated metabolism, the pentose phosphate pathway, biofilm formation (*Vibrio cholerae*), bacterial chemotaxis, and flagellar assembly. Central energy- and carbon-metabolism pathways, including the citrate cycle and oxidative phosphorylation, show a modest but consistent *Rugulopteryx*-leaning skew after renormalization rather than forming a shared functional backbone; high-abundance pathways that are closely balanced in this dataset instead include purine metabolism, quorum sensing, and pyrimidine metabolism (Section 9.5). The ecological-context illustration is conceptual; the pathway differences shown represent metagenomic functional potential rather than measurements of pathway activity.

**Figure 3:**
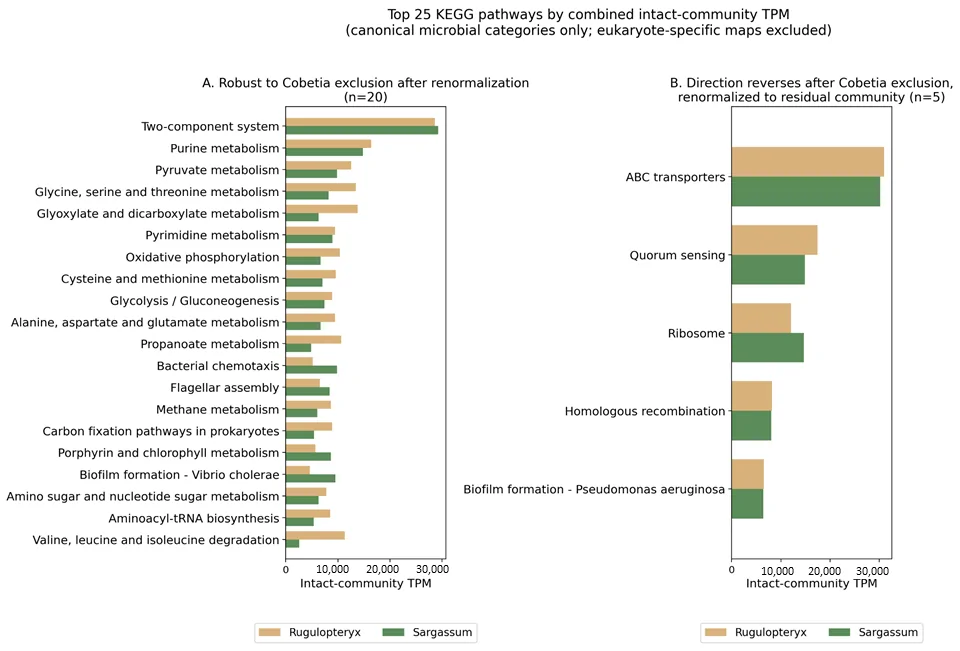
Top 25 KEGG pathways by combined intact-community TPM, restricted to canonical microbial top-level categories (Metabolism, Genetic Information Processing, Environmental Information Processing, Cellular Processes) with eukaryote-, plant-, and animal-specific pathway maps additionally excluded (162 canonical microbial pathways total; see Supplementary Methods), and partitioned according to sensitivity to *Cobetia* exclusion. TPM values shown are intact-community values for both panels. (**A**) Twenty pathways retain the same direction of the *Rugulopteryx*–*Sargassum* sp. contrast after *Cobetia*-assigned ORFs are excluded and their post-exclusion TPM is renormalized to the residual community total. (**B**) Five pathways change direction under the same renormalized comparison: ABC transporters, quorum sensing, ribosome, homologous recombination, and biofilm formation (*Pseudomonas aeruginosa*). The two panels distinguish directionally retained and *Cobetia*-sensitive pathway contrasts rather than genuine and artefactual biological functions.

**Figure 4:**
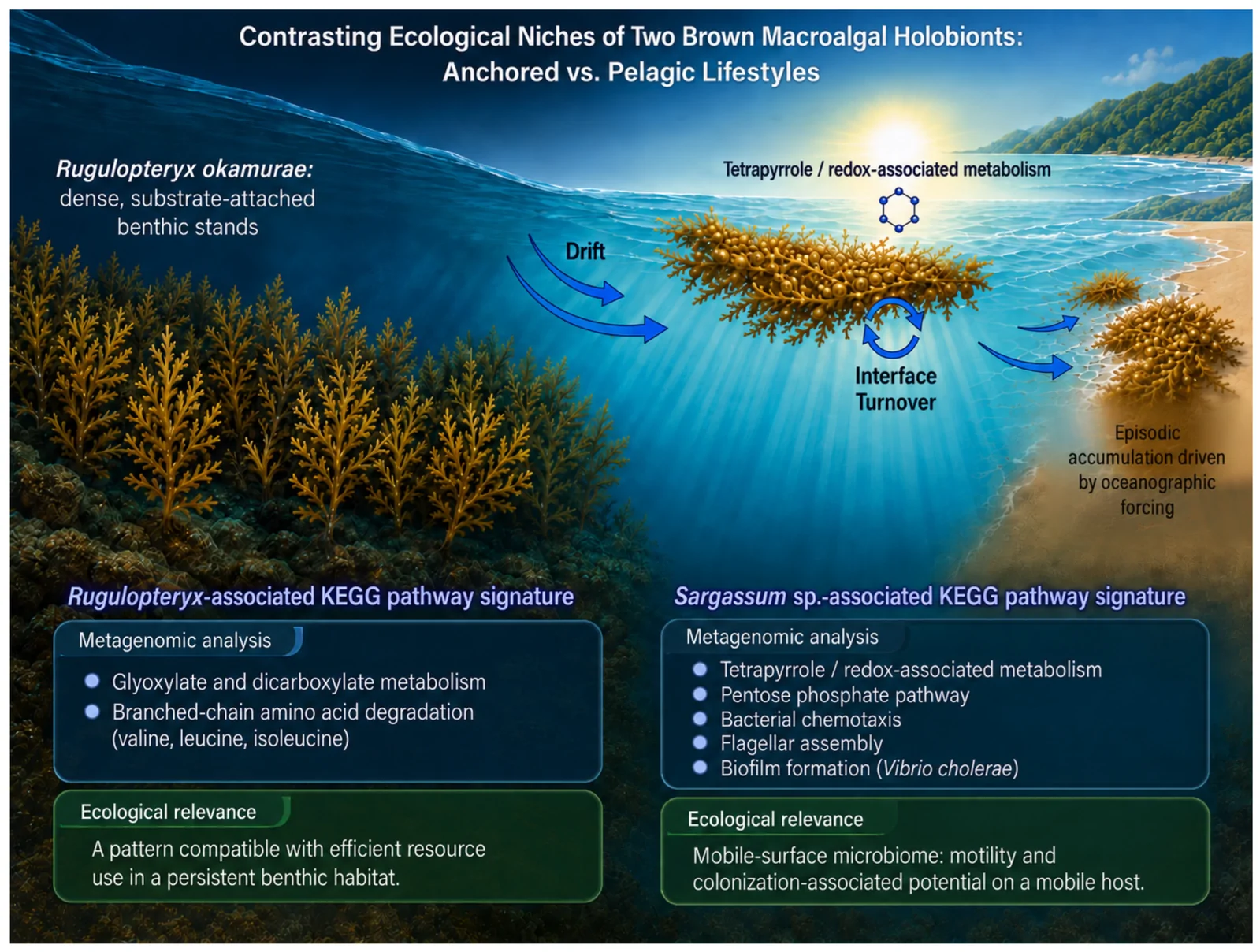
Ecological-functional synthesis of the *Rugulopteryx okamurae* and *Sargassum* sp. microbiome profiles. The *Rugulopteryx*-associated microbiome retains greater representation of glyoxylate/dicarboxylate metabolism and branched-chain amino-acid degradation after exclusion of *Cobetia*-assigned ORFs, consistent with comparatively strong resource-recycling potential in a persistent benthic environment. The *Sargassum* sp.-associated microbiome retains greater representation of the pentose phosphate pathway, tetrapyrrole-associated metabolism, bacterial chemotaxis, flagellar assembly, and biofilm formation (*Vibrio cholerae*), consistent with redox-associated metabolism and microbial surface-colonization potential on a mobile host. These associations derive from metagenomic functional potential and do not demonstrate microbiome-mediated benthic dominance, raft cohesion, or long-distance dispersal.

**Table 1:** Evidence supporting major metabolic and stress-associated functions proposed for macroalgal microbiomes.

Process	Evidence Currently Available	Ecological Interpretation	Evidence Strength
Host-derived carbon utilization	Macroalgal DOM release; ABC/TRAP and other substrate-transport genes in associated bacteria [4,26,27,28]	Host-associated organic compounds provide substrates that can structure heterotrophic surface communities	Strong ecological basis; genomic support for microbial utilization
Vitamin B12 biosynthesis	Bacterial cobalamin-biosynthesis pathways; experimentally demonstrated microbial B12 provisioning in selected algal systems [4,29,30]	Associated bacteria may supply B12 to physiologically dependent algal hosts	Experimentally demonstrated in selected algal–bacterial systems; direct transfer within individual macroalgal holobionts remains incompletely established.
Nitrogen transformations	Genes for nitrate/nitrite reduction, urea hydrolysis, and ammonium metabolism in macroalgal-associated metagenomes; experimentally demonstrated nitrogen fixation and nitrogen provisioning to *Ulva prolifera* by *Cobetia* isolates in a defined co-culture system [4,31].	Microbial nitrogen metabolism can alter local nitrogen speciation, and in at least one experimentally resolved system nitrogen-fixing bacteria can directly contribute biologically available nitrogen to the algal host.	Mixed: genomic support for several nitrogen transformations; direct experimental evidence for nitrogen fixation, host provisioning, and host growth response in an Ulva–Cobetia system.
DMSP demethylation	*dmdA* and downstream demethylation pathways [34]	Microbial utilization of host-associated DMSP and sulfur assimilation	Strong biochemical and genomic basis for microbial DMSP metabolism; host-specific ecological consequences remain context dependent.
DMSP cleavage	Ddd-family lyases and related cleavage systems [36,37]	DMS production and marine sulfur cycling	Strong biochemical basis for DMS-producing cleavage pathways; their quantitative importance within individual macroalgal holobionts requires direct measurement.
Cold-associated developmental support	Cold-adapted bacterial isolates retain morphogenesis-promoting effects in *Ulva* [38]	Microbial developmental function can persist under polar thermal conditions	Experimental
Low-salinity acclimation	Microbiome perturbation compromises acclimation of *Ectocarpus subulatus* [39]	Associated microorganisms contribute to host salinity tolerance	Experimental; mechanism incompletely resolved
Metal resistance and transformation	Efflux, sequestration, EPS binding, redox transformation and volatilization mechanisms in marine bacteria and algal–bacterial consortia [40]	Microorganisms may alter metal speciation and potentially reduce local exposure	Microbial capability supported; direct host protection less well established

**Table 2:** Comparison of sequence-based and function-based approaches for metagenomic discovery in macroalgal microbiomes.

Feature	Sequence-Based Metagenomic Mining	Function-Based Metagenomic Mining
Primary principle	Detection of genes or pathways through sequence similarity, conserved domains, genomic context, and computational prediction	Detection of biological activity through expression of environmental DNA in a heterologous host
Typical tools	HMM profiles, curated functional databases, dbCAN, antiSMASH, comparative genomics [54,55]	Plasmid, cosmid, fosmid or BAC libraries; enzymatic or phenotype-based screening [41,42,57]
Principal strength	High throughput; scalable; preserves genomic context; suitable for genome- and pathway-level discovery	Can identify activities with weak or no recognizable similarity to characterized genes
Principal limitation	Strongly dependent on reference databases and annotation accuracy [45,56]	Dependent on successful transcription, translation, folding, cofactor supply and, where relevant, secretion in the heterologous host [57,58]
Ecological inference	Identifies encoded functional potential but does not establish activity [6,7]	Demonstrates activity in the screening host but not necessarily ecological activity in the original community [57,58]
Macroalgal applications	Identification of CAZymes, PULs, transport systems, BGCs, stress-response pathways and taxon-specific metabolic repertoires [4,54,55]	Screening for alginate lyases, agarases, carrageenases, Ulvan-degrading enzymes, lipases, antimicrobials and other detectable activities [41,42,57]
Appropriate endpoint	Candidate gene, pathway, MAG or BGC for subsequent validation [53,56,59]	Experimentally detected activity requiring gene identification and biochemical characterization [57,59]
Strongest use	Large-scale discovery and taxon-resolved prioritization [53,56]	Discovery and validation of previously unrecognized biochemical activities [57,58]

**Table 3:** Major macroalgal glycan classes and microbial systems associated with their degradation.

Glycan Class	Principal Algal Source	Candidate Microbial Enzyme Systems	Representative Associated Taxa	Level of Functional Inference
Alginate	Brown algae	Alginate lyases, including PL-family enzymes involved in polymer and oligoalginate cleavage	Saprospiraceae, Maricaulaceae, *Granulosicoccus* and other marine bacteria [4,61]	Genes and characterized enzymes available; substrate activity varies among taxa
Laminarin	Brown-algal storage glucan	β-1,3-glucanases and accessory enzymes acting on branched β-glucans	Flavobacteriaceae, Cytophagaceae and other marine heterotrophs [61]	Well-established microbial substrate; exact activity requires enzyme- or strain-level confirmation
*Ulva*n	Green algae, particularly *Ulva*	*Ulva*n lyases, glycoside hydrolases, sulfatases and accessory enzymes	*Zobellia*, *Maribacter* and related Bacteroidota [62]	Specialized systems characterized in selected taxa; metagenomic predictions require validation
Fucoidans and related sulfated glycans	Brown algae	Sulfatases, glycoside hydrolases and other carbohydrate-processing enzymes	Bacteroidota, Verrucomicrobiota, including some Akkermansiaceae [4]	Large enzymatic repertoires indicate potential; precise substrate specificity often unresolved
Agarans	Red algae	Agarases and associated glycoside hydrolases	Diverse marine Bacteroidota and Gammaproteobacteria [65]	Multiple characterized enzymes available; community-level prediction remains substrate-dependent
Carrageenans	Red algae	Carrageenases, sulfatases and accessory enzymes	Specialized marine heterotrophic bacteria [63,65]	Biochemical activity demonstrated in selected isolates; metagenomic candidates require confirmation
Other sulfated/complex polysaccharides	Multiple macroalgal lineages	Combined GH, PL, CE, sulfatase and transport systems	Taxonomically diverse marine bacteria [55]	Functional potential often inferred from gene combinations and PUL architecture

**Table 4:** Examples of biotechnologically relevant functions and metabolites associated with macroalgal microbiomes.

Functional Category	Example	Microbial Source/Context	Evidence Currently Available	Translational Status
Antimicrobial natural product	Kocumarin	*Kocuria marina* associated with *Pelvetia canaliculata*	Compound isolated and antimicrobial activity demonstrated [67]	Early-stage bioactive lead
Pigmented bioactive metabolite	Decylprodigiosin	*Streptomyces violaceoruber* associated with *Codium tomentosum*	Compound isolated and biological activity characterized [68]	Early-stage natural-product lead
Morphogen	Thallusin	Marine bacterial associates of green macroalgae	Chemically characterized; developmental effects experimentally demonstrated [14,15,16]	Strong biological validation; application potential under investigation
Antifungal/biocontrol activity	Metabolites from *Bacillus halotolerans*	Endophyte associated with *Sargassum wightii*	Antagonistic activity against *Fusarium incarnatum* reported [71]	Early-stage agricultural biocontrol candidate
Quorum-signaling molecules	AHLs	Seaweed-associated Gram-negative bacteria	Production documented in selected strains; broader crop applications inferred from plant literature [66,70]	Mechanistically plausible; application-specific validation required
Polymer-derived elicitors	Alginate/chitin-derived oligosaccharides	Generated through enzymatic degradation of marine polymers	Biological elicitor activity demonstrated in plant systems [66]	Application potential depends on defined production and formulation
Biosynthetic gene clusters	NRPS, PKS, RiPP, terpene-associated clusters	Metagenomes and MAGs of seaweed-associated bacteria	Computational prediction [53,54]	Discovery stage until metabolite production is demonstrated
Heterologously expressed pathways	Candidate marine BGCs	Reconstructed in laboratory chassis organisms	Technically feasible in selected systems [53,60]	Highly pathway-dependent; requires optimization

**Table 5:** Comparison of major evidence types across algal microbiome systems.

Research Dimension	Macroalgal Systems	Microalgal Systems	Cyanobacterial/Extremophilic Systems	Principal Limitation
Host-associated community structure	Extensive evidence across multiple seaweed hosts	Extensive in cultured and natural phycospheres	Extensive in natural and engineered communities	Taxonomic association does not establish function [6,7]
Experimentally demonstrated developmental interaction	Particularly strong in *Ulva*–bacteria models	Growth-promoting interactions demonstrated in selected systems	Less directly comparable	Strong mechanistic evidence concentrated in few models [12,13]
Vitamin and micronutrient exchange	Genomically supported; direct transfer demonstrated unevenly	Strong experimental precedents, particularly for B12 and iron-related interactions	Variable among systems	Host dependence and transfer often system-specific [29,74]
Genome-resolved microbiome analysis	Strong in selected kelp and other macroalgal systems	Growing but uneven	Increasingly extensive in environmental metagenomics	MAG quality and activity remain separate issues [4]
Glycan-degrading specialization	Particularly well developed because of lineage-specific macroalgal polysaccharides	Less central to host biology	Variable	Substrate specificity cannot be inferred reliably from sequence alone [61,64]
Natural-product genome mining	Growing field; comparatively few fully validated metagenomic examples	Increasing	Highly developed in selected cyanobacterial systems	BGC prediction does not establish metabolite production [54,79]
Synthetic or managed consortia	Emerging, especially for aquaculture	Comparatively well developed in bioreactor systems	Used in selected biotechnology applications	Laboratory stability may not translate to open environments [73,76]
Environmental acclimation	Experimental evidence in selected systems such as *Ectocarpus* and *Ulva*	Extensive observational and experimental literature	Strongly context dependent	Community change does not necessarily imply microbial causality [38,39]
Industrial translation	Mostly discovery to early validation	Some technologies reach pilot or operational scales	Product-specific and highly variable	Translation should be evaluated case by case rather than inferred from genomic potential [59,73]

## Data Availability

The shotgun metagenomic sequence data generated in this study for the *Rugulopteryx okamurae* and *Sargassum* sp. holobionts have been deposited in the NCBI Sequence Read Archive (SRA) under BioProject accession PRJNA1521022. Raw paired-end sequencing reads are available under BioSample accessions SAMN62786275 (Rugu-Meta-N2033, *Rugulopteryx okamurae*) and SAMN62786276 (Sarg-Meta-N2034, *Sargassum* sp.), corresponding to SRA run accessions SRR40416375 and SRR40416374, respectively. The complete processed data and analysis code underlying Section 9, including the full pathway tables and all analysis scripts, will be additionally available via the preprint record upon resubmission of the revised version of this manuscript.

## Supplementary Materials

The following supporting information can be downloaded at: https://www.mdpi.com/article/10.3390/microorganisms14102127/s1, Table S1: Full canonical microbial KEGG pathway table (162 pathways), intact-community TPM; Table S2: All-pathway balance ranking before/after *Cobetia* exclusion, including renormalized and non-renormalized comparisons; Table S3: Reanalysis of the original MICROBIOTECH 2025 pathway categorization, including renormalized comparison; Table S4: Taxonomic composition at class level; Table S5: *Cobetia* contig-level distribution; Table S6: *Cobetia* KEGG functional profile; Code: Analysis scripts and documentation, including the eukaryotic-pathway-map exclusion list.

## References

[1] Egan S., Harder T., Burke C., Steinberg P., Kjelleberg S., Thomas T. (2013). The seaweed holobiont: Understanding seaweed–bacteria interactions. FEMS Microbiol. Rev..

[2] Florez J.Z., Camus C., Hengst M.B., Buschmann A.H. (2017). A Functional Perspective Analysis of Macroalgae and Epiphytic Bacterial Community Interaction. Front. Microbiol..

[3] Ghaderiardakani F., Quartino M.L., Wichard T. (2020). Microbiome-dependent adaptation of seaweeds under environmental stresses: A perspective. Front. Mar. Sci..

[4] Weigel B.L., Miranda K.K., Fogarty E.C., Watson A.R., Pfister C.A. (2022). Functional insights into the kelp microbiome from metagenome-assembled genomes. mSystems.

[5] Marzinelli E.M., Thomas T., Vadillo Gonzalez S., Egan S., Steinberg P.D. (2024). Seaweeds as holobionts: Current state, challenges, and potential applications. J. Phycol..

[6] Quince C., Walker A.W., Simpson J.T., Loman N.J., Segata N. (2017). Shotgun metagenomics, from sampling to analysis. Nat. Biotechnol..

[7] Franzosa E.A., Hsu T., Sirota-Madi A., Shafquat A., Abu-Ali G., Morgan X.C., Huttenhower C. (2015). Sequencing and beyond: Integrating molecular ‘omics’ for microbial community profiling. Nat. Rev. Microbiol..

[8] Nayfach S., Pollard K.S. (2016). Toward accurate and quantitative comparative metagenomics. Cell.

[9] Zilber-Rosenberg I., Rosenberg E. (2008). Role of microorganisms in the evolution of animals and plants: The hologenome theory of evolution. FEMS Microbiol. Rev..

[10] Califano G., Kwantes M., Abreu M.H., Costa R., Wichard T. (2020). Cultivating the macroalgal holobiont: Effects of integrated multi-trophic aquaculture on the microbiome of *Ulva rigida* (Chlorophyta). Front. Mar. Sci..

[11] Wang J., Tang X., Mo Z., Mao Y. (2022). Metagenome-assembled genomes from *Pyropia haitanensis* microbiome provide insights into the potential metabolic functions to the seaweed. Front. Microbiol..

[12] Spoerner M., Wichard T., Bachhuber T., Stratmann J.O., Oertel W. (2012). Growth and thallus morphogenesis of *Ulva mutabilis* (Chlorophyta) depends on a combination of two bacterial species excreting regulatory factors. J. Phycol..

[13] Ghaderiardakani F., Coates J.C., Wichard T. (2017). Bacteria-induced morphogenesis of *Ulva intestinalis* and *Ulva mutabilis* (Chlorophyta): A contribution to the lottery theory. FEMS Microbiol. Ecol..

[14] Matsuo Y., Imagawa H., Nishizawa M., Shizuri Y. (2005). Isolation of an algal morphogenesis inducer from a marine bacterium. Science.

[15] Alsufyani T., Califano G., Deicke M., Grueneberg J., Weiss A., Engelen A.H., Kwantes M., Mohr J.F., Ulrich J.F., Wichard T. (2020). Macroalgal–bacterial interactions: Identification and role of thallusin in morphogenesis of the seaweed *Ulva* (Chlorophyta). J. Exp. Bot..

[16] Dhiman S., Ulrich J.F., Wienecke P., Wichard T., Arndt H.-D. (2022). Stereoselective total synthesis of (−)-thallusin for bioactivity profiling. Angew. Chem. Int. Ed..

[17] Saha M., Weinberger F. (2019). Microbial “gardening” by a seaweed holobiont: Surface metabolites attract protective and deter pathogenic epibacterial settlement. J. Ecol..

[18] Egan S., Fernandes N.D., Kumar V., Gardiner M., Thomas T. (2014). Bacterial pathogens, virulence mechanisms and host defense in marine macroalgae. Environ. Microbiol..

[19] Abdelrazek H.M., Shams El-Din N.G., Ghozlan H.A., Sabry S.A., Abouelkheir S.S. (2024). Distribution and functional perspective analysis of epiphytic and endophytic bacterial communities associated with marine seaweeds, Alexandria shores, Egypt. BMC Microbiol..

[20] Quéré G., Intertaglia L., Payri C., Galand P.E. (2019). Disease Specific Bacterial Communities in a Coralline Algae of the Northwestern Mediterranean Sea: A Combined Culture Dependent and -Independent Approach. Front. Microbiol..

[21] Yang Z., Sun T., Chen J., Wang J., Yu X., Zhao Y., Li Y., Tang X., Xiao H. (2023). The contribution of host tissue location and sex to epiphytic bacterial community assembly of *Sargassum thunbergii*. Ecol. Indic..

[22] Lemay M.A., Martone P.T., Hind K.R., Lindstrom S.C., Parfrey L.W. (2018). Alternate life history phases of a common seaweed have distinct microbial surface communities. Mol. Ecol..

[23] Veenhof R.J., McGrath A.H., Champion C., Dworjanyn S.A., Marzinelli E.M., Coleman M.A. (2025). The role of microbiota in kelp gametophyte development and resilience to thermal stress. J. Phycol..

[24] Trias R., García-Lledó A., Sánchez N., López-Jurado J.L., Hallin S., Bañeras L. (2012). Abundance and composition of epiphytic bacterial and archaeal ammonia oxidizers of marine red and brown macroalgae. Appl. Environ. Microbiol..

[25] Zhao J., Nair S., Zhang Z., Wang Z., Jiao N., Zhang Y. (2024). Macroalgal virosphere assists with host-microbiome equilibrium regulation and affects prokaryotes in surrounding marine environments. ISME J..

[26] Wada S., Aoki M.N., Tsuchiya Y., Sato T., Shinagawa H., Hama T. (2007). Quantitative and qualitative analyses of dissolved organic matter released from *Ecklonia cava* Kjellman, in Oura Bay, Shimoda, Izu Peninsula, Japan. J. Exp. Mar. Biol. Ecol..

[27] Reed D.C., Carlson C.A., Halewood E.R., Nelson J.C., Harrer S.L., Rassweiler A., Miller R.J. (2015). Patterns and controls of reef-scale production of dissolved organic carbon by giant kelp *Macrocystis pyrifera*. Limnol. Oceanogr..

[28] Weigel B.L., Pfister C.A. (2021). The dynamics and stoichiometry of dissolved organic carbon release by kelp. Ecology.

[29] Croft M.T., Lawrence D.A., Raux-Deery E., Warren M.J., Smith A.G. (2005). Algae acquire vitamin B12 through a symbiotic relationship with bacteria. Nature.

[30] Helliwell K.E., Wheeler G.L., Leptos K.C., Goldstein R.E., Smith A.G. (2011). Insights into the evolution of vitamin B12 auxotrophy from sequenced algal genomes. Mol. Biol. Evol..

[31] Wu T., Bi F., Liu H., Wang S., He P., Zhang J. (2025). Identification of nitrogen-fixing bacteria on green tide–causing species and evaluation of their nitrogen-fixing capacity. Bioresour. Technol..

[32] Stefels J. (2000). Physiological aspects of the production and conversion of DMSP in marine algae and higher plants. J. Sea Res..

[33] Sunda W., Kieber D.J., Kiene R.P., Huntsman S. (2002). An antioxidant function for DMSP and DMS in marine algae. Nature.

[34] Howard E.C., Henriksen J.R., Buchan A., Reisch C.R., Bürgmann H., Welsh R., Ye W., González J.M., Mace K., Joye S.B. (2006). Bacterial taxa that limit sulfur flux from the ocean. Science.

[35] Seymour J.R., Simó R., Ahmed T., Stocker R. (2010). Chemoattraction to dimethylsulfoniopropionate throughout the marine microbial food web. Science.

[36] Todd J.D., Rogers R., Li Y.G., Wexler M., Bond P.L., Sun L., Curson A.R.J., Malin G., Steinke M., Johnston A.W.B. (2007). Structural and regulatory genes required to make the gas dimethyl sulfide in bacteria. Science.

[37] Alcolombri U., Ben-Dor S., Feldmesser E., Levin Y., Tawfik D.S., Vardi A. (2015). Identification of the algal dimethyl sulfide-releasing enzyme: A missing link in the marine sulfur cycle. Science.

[38] Ghaderiardakani F., Ulrich J.F., Barth E., Quartino M.L., Wichard T. (2025). Algal growth and morphogenesis-promoting factors released by cold-adapted bacteria contribute to the resilience and morphogenesis of the seaweed *Ulva* (Chlorophyta) in Antarctica (Potter Cove). J. Plant Growth Regul..

[39] Dittami S.M., Duboscq-Bidot L., Perennou M., Gobet A., Corre E., Boyen C., Tonon T. (2016). Host–microbe interactions as a driver of acclimation to salinity gradients in brown algal cultures. ISME J..

[40] Peña-Salamanca E.J., Rengifo-Gallego A.L., Benitez-Campo N., Kim S.-K. (2011). Detoxification mechanisms of heavy metals by algal–bacteria consortia. Handbook of Marine Macroalgae: Biotechnology and Applied Phycology.

[41] Berini F., Casciello C., Marcone G.L., Marinelli F. (2017). Metagenomics: Novel enzymes from non-culturable microbes. FEMS Microbiol. Lett..

[42] Kennedy J., Marchesi J.R., Dobson A.D.W. (2008). Marine metagenomics: Strategies for the discovery of novel enzymes with biotechnological applications from marine environments. Microb. Cell Fact..

[43] Burke C., Kjelleberg S., Thomas T. (2009). Selective extraction of bacterial DNA from the surfaces of macroalgae. Appl. Environ. Microbiol..

[44] Taberlet P., Coissac E., Hajibabaei M., Rieseberg L.H. (2012). Environmental DNA. Mol. Ecol..

[45] Prakash T., Taylor T.D. (2012). Functional assignment of metagenomic data: Challenges and applications. Brief. Bioinform..

[46] Tyson G.W., Chapman J., Hugenholtz P., Allen E.E., Ram R.J., Richardson P.M., Solovyev V.V., Rubin E.M., Rokhsar D.S., Banfield J.F. (2004). Community structure and metabolism through reconstruction of microbial genomes from the environment. Nature.

[47] Alneberg J., Bjarnason B.S., de Bruijn I., Schirmer M., Quick J., Ijaz U.Z., Lahti L., Loman N.J., Andersson A.F., Quince C. (2014). Binning metagenomic contigs by coverage and composition. Nat. Methods.

[48] Parks D.H., Imelfort M., Skennerton C.T., Hugenholtz P., Tyson G.W. (2015). CheckM: Assessing the quality of microbial genomes recovered from isolates, single cells, and metagenomes. Genome Res..

[49] Tettelin H., Masignani V., Cieslewicz M.J., Donati C., Medini D., Ward N.L., Angiuoli S.V., Crabtree J., Jones A.L., Durkin A.S. (2005). Genome analysis of multiple pathogenic isolates of *Streptococcus agalactiae*: Implications for the microbial “pan-genome”. Proc. Natl. Acad. Sci. USA.

[50] McInerney J.O., McNally A., O’Connell M.J. (2017). Why prokaryotes have pangenomes. Nat. Microbiol..

[51] Shapiro B.J., Friedman J., Cordero O.X., Preheim S.P., Timberlake S.C., Szabó G., Polz M.F., Alm E.J. (2012). Population genomics of early events in the ecological differentiation of bacteria. Science.

[52] Hunt D.E., David L.A., Gevers D., Preheim S.P., Alm E.J., Polz M.F. (2008). Resource partitioning and sympatric differentiation among closely related bacterioplankton. Science.

[53] Trindade M., van Zyl L.J., Navarro-Fernández J., Abd Elrazak A. (2015). Targeted metagenomics as a tool to tap into marine natural product diversity for the discovery and production of drug candidates. Front. Microbiol..

[54] Blin K., Shaw S., E Augustijn H., Reitz Z.L., Biermann F., Alanjary M., Fetter A., Terlouw B.R., Metcalf W.W., Helfrich E.J.N. (2023). antiSMASH 7.0: New and improved predictions for detection, regulation, chemical structures and visualisation. Nucleic Acids Res..

[55] Zheng J., Ge Q., Yan Y., Zhang X., Huang L., Yin Y. (2023). dbCAN3: Automated carbohydrate-active enzyme and substrate annotation. Nucleic Acids Res..

[56] Medema M.H., Fischbach M.A. (2015). Computational approaches to natural product discovery. Nat. Chem. Biol..

[57] Uchiyama T., Miyazaki K. (2009). Functional metagenomics for enzyme discovery: Challenges to efficient screening. Curr. Opin. Biotechnol..

[58] Ekkers D.M., Cretoiu M.S., Kielak A.M., van Elsas J.D. (2012). The great screen anomaly—A new frontier in product discovery through functional metagenomics. Appl. Microbiol. Biotechnol..

[59] Ferrer M., Martínez-Martínez M., Bargiela R., Streit W.R., Golyshina O.V., Golyshin P.N. (2016). Estimating the success of enzyme bioprospecting through metagenomics: Current status and future trends. Microb. Biotechnol..

[60] Schmidt E.W., Nelson J.T., Rasko D.A., Sudek S., Eisen J.A., Haygood M.G., Ravel J. (2005). Patellamide A and C biosynthesis by a microcin-like pathway in *Prochloron didemni*, the cyanobacterial symbiont of *Lissoclinum patella*. Proc. Natl. Acad. Sci. USA.

[61] Kabisch A., Otto A., König S., Becher D., Albrecht D., Schüler M., Teeling H. (2014). Functional characterization of polysaccharide utilization loci in the marine *Bacteroidetes “Gramella forsetii”* KT0803. ISME J..

[62] Salinas A., French C.E. (2017). The enzymatic *Ulva*n depolymerisation system from the alga-associated marine flavobacterium *Formosa agariphila*. Algal Res..

[63] Ficko-Blean E., Préchoux A., Thomas F., Rochat T., Larocque R., Zhu Y., Stam M., Génicot S., Jam M., Calteau A. (2017). Carrageenan catabolism is encoded by a complex regulon in marine heterotrophic bacteria. Nat. Commun..

[64] Lu D.C., Wang F.Q., Amann R.I., Teeling H., Du Z.J. (2023). Epiphytic common core bacteria in the microbiomes of co-located green (*Ulva*), brown (*Saccharina*) and red (*Grateloupia*, *Gelidium*) macroalgae. Microbiome.

[65] Schultz-Johansen M., Bech P.K., Hennessy R.C., Glaring M.A., Barbeyron T., Czjzek M., Stougaard P. (2018). A novel enzyme portfolio for red algal polysaccharide degradation in the marine bacterium *Paraglaciecola hydrolytica* S66T encoded in a sizeable polysaccharide utilization locus. Front. Microbiol..

[66] McKenna S.M., Da Silva Pereira E.H., Fort A. (2025). Seaweed-associated microbes as a novel source of crop agrochemicals. Front. Mar. Sci..

[67] Uzair B., Menaa F., Khan B.A., Mohammad F.V., Ahmad V.U., Djeribi R., Menaa B. (2018). Isolation, purification, structural elucidation and antimicrobial activities of kocumarin, a novel antibiotic isolated from actinobacterium *Kocuria marina* CMG S2 associated with the brown seaweed *Pelvetia canaliculata*. Microbiol. Res..

[68] Girão M., Freitas S., Martins T.P., Urbatzka R., Carvalho M.F., Leão P.N. (2024). Decylprodigiosin: A new member of the prodigiosin family isolated from a seaweed-associated Streptomyces. Front. Pharmacol..

[69] Romano S., Jackson S.A., Patry S., Dobson A.D.W. (2018). Extending the “One Strain Many Compounds” (OSMAC) principle to marine microorganisms. Mar. Drugs.

[70] Aslam M., Pei P., Ye P., Li T., Liang H., Zhang Z., Ke X., Chen W., Du H. (2023). Unraveling the diverse profile of N-acyl homoserine lactone signals and their role in the regulation of biofilm formation in *Porphyra haitanensis*-associated *Pseudoalteromonas galatheae*. Microorganisms.

[71] Suji H.A., Manikandan K., Sudha A., Muthukumar A., Jeyalakshmi C., Charumathi M., Rajesh M., Raj T.S. (2024). Whole genome sequence of seaweed endophyte *Bacillus halotolerans* strain AUPP for antagonistic activity against *Fusarium incarnatum* causing chilli fruit rot. Sci. Rep..

[72] González-González L.M., de-Bashan L.E. (2021). Toward the enhancement of microalgal metabolite production through microalgae–bacteria consortia. Biology.

[73] Tong C.Y., Honda K., Derek C.J.C. (2023). A review on microalgal-bacterial co-culture: The multifaceted role of beneficial bacteria towards enhancement of microalgal metabolite production. Environ. Res..

[74] Amin S.A., Green D.H., Hart M.C., Küpper F.C., Sunda W.G., Carrano C.J. (2009). Photolysis of iron–siderophore chelates promotes bacterial–algal mutualism. Proc. Natl. Acad. Sci. USA.

[75] Amin S.A., Hmelo L.R., Van Tol H.M., Durham B.P., Carlson L.T., Heal K.R., Morales R.L., Berthiaume C.T., Parker M.S., Djunaedi B. (2015). Interaction and signaling between a cosmopolitan phytoplankton and associated bacteria. Nature.

[76] Subashchandrabose S.R., Ramakrishnan B., Megharaj M., Venkateswarlu K., Naidu R. (2011). Consortia of cyanobacteria/microalgae and bacteria: Biotechnological potential. Biotechnol. Adv..

[77] Patel A., Matsakas L., Rova U., Christakopoulos P. (2019). A perspective on biotechnological applications of thermophilic microalgae and cyanobacteria. Bioresour. Technol..

[78] Singh R., Parihar P., Singh M., Bajguz A., Kumar J., Singh S., Singh V.P., Prasad S.M. (2017). Uncovering potential applications of cyanobacteria and algal metabolites in biology, agriculture and medicine: Current status and future prospects. Front. Microbiol..

[79] Zammit G., Zammit M.G., Buttigieg K.G. (2023). Emerging technologies for the discovery of novel diversity in cyanobacteria and algae and the elucidation of their valuable metabolites. Diversity.

[80] Vila Duplá M. (2025). Advancements in algal microbiome research: A game-changer for climate resilience and invasion success?. Microb. Ecol..

[81] Bonthond G., Bayer T., Krueger-Hadfield S.A., Stärck N., Wang G., Nakaoka M., Künzel S., Weinberger F. (2021). The role of host promiscuity in the invasion process of a seaweed holobiont. ISME J..

[82] Mei X., Wu C., Zhao J., Yan T., Jiang P. (2019). Community structure of bacteria associated with drifting *Sargassum horneri*, the causative species of golden tide in the Yellow Sea. Front. Microbiol..

[83] Zang Y., Liu X., Xue S., Yin L., Fan S., Miao X., Fu M., Xiao J., Wang Z. (2025). A comparative analysis of bacterial community characterization and host–bacteria interactions between bi-macroalgal blooms caused by *Ulva prolifera* and *Sargassum horneri*. Front. Microbiol..

[84] Aires T., Stuij T.M., Muyzer G., Serrão E.A., Engelen A.H. (2021). Characterization and comparison of bacterial communities of an invasive and two native *Caribbean seagrass* species sheds light on the possible influence of the microbiome on invasive mechanisms. Front. Microbiol..

[85] Bettencourt R., Moreira J. (2025). KEGG-resolved microbiome contrasts in invasive brown-algal holobionts reveal complementary bioprocessing pathways and actionable mitigation strategies. Proceedings of the MICROBIOTECH 2025, Ponta Delgada, Portugal, 4–6 December 2025.

[86] Laamraoui M.R., Mghili B., Roca M., Chaieb O., Ostalé-Valriberas E., Martín-Zorrilla A., Sabino-Lorenzo A., Aarab S. (2024). Rapid invasion and expansion of the invasive macroalgae *Rugulopteryx okamurae* in the Mediterranean and Atlantic: A 10-year review. Mar. Pollut. Bull..

[87] Johns E.M., Lumpkin R., Putman N.F., Smith R.H., Muller-Karger F.E., Rueda-Roa D.T., Hu C., Wang M., Brooks M.T., Gramer L.J. (2020). The establishment of a pelagic Sargassum population in the tropical Atlantic: Biological consequences of a basin-scale long distance dispersal event. Prog. Oceanogr..

[88] van Hees D.H., Olsen Y.S., Mattio L., Ruiz-Montoya L., Wernberg T., Kendrick G.A. (2019). Cast adrift: Physiology and dispersal of benthic *Sargassum spinuligerum* in surface rafts. Limnol. Oceanogr..

[89] Tamames J., Puente-Sánchez F. (2019). SqueezeMeta, a highly portable, fully automatic metagenomic analysis pipeline. Front. Microbiol..

[90] Schmelz P., Eckensperger S., Osvatic J., Séneca J., Alzubaidy H., Petersen J.M. (2026). Host depletion kits improve microbiome analyses in environmental samples: Seagrass as a test case. ISME Commun..

[91] Gloor G.B., Macklaim J.M., Pawlowsky-Glahn V., Egozcue J.J. (2017). Microbiome Datasets Are Compositional: And This Is Not Optional. Front. Microbiol..

[92] Mattio L., Payri C.E. (2011). 190 Years of Sargassum Taxonomy, Facing the Advent of DNA Phylogenies. Bot. Rev..

[93] González-Nieto D., Oliveira M.C., Resendiz M.L.N., Dreckmann K.M., Mateo-Cid L.E., Sentíes A. (2020). Molecular assessment of the genus *Sargassum* (Fucales, Phaeophyceae) from the Mexican coasts of the Gulf of Mexico and Caribbean, with the description of *S. xochitlae* sp. nov. Phytotaxa.

[94] Ensign S.A. (2006). Revisiting the glyoxylate cycle: Alternate pathways for microbial acetate assimilation. Mol. Microbiol..

[95] Massey L.K., Sokatch J.R., Conrad R.S. (1976). Branched-chain amino acid catabolism in bacteria. Bacteriol. Rev..

[96] Stincone A., Prigione A., Cramer T., Wamelink M.M.C., Campbell K., Cheung E., Olin-Sandoval V., Grüning N.-M., Krüger A., Tauqeer Alam M. (2015). The return of metabolism: Biochemistry and physiology of the pentose phosphate pathway. Biol. Rev..

[97] Layer G. (2021). Heme biosynthesis in prokaryotes. Biochim. Biophys. Acta Mol. Cell Res..

[98] Hengge R. (2009). Principles of c-di-GMP signaling in bacteria. Nat. Rev. Microbiol..

[99] Lelchat F., Cérantola S., Brandily C., Colliec-Jouault S., Baudoux A.-C., Ojima T., Boisset C. (2015). The marine bacteria *Cobetia marina* DSMZ 4741 synthesizes an unexpected K-antigen-like exopolysaccharide. Carbohydr. Polym..

[100] Amirinejad N., Mohammadi M., Shekarchizadeh A., Askarinejad Behzadi M., Hassanshahian M., Ataie S.A., Hjazi A., Jabber Ibrahim A. (2024). Isolation and characterization of glycolipid biosurfactant produced by marine bacterium *Cobetia marina* strain F1 and investigation of antimicrobial and anti-biofilm activity. Geomicrobiol. J..

[101] Ibacache-Quiroga C., Ojeda J., Espinoza-Vergara G., Olivero P., Cuellar M., Dinamarca M.A. (2013). The hydrocarbon-degrading marine bacterium *Cobetia* sp. strain MM1IDA2H-1 produces a biosurfactant that interferes with quorum sensing of fish pathogens by signal hijacking. Microb. Biotechnol..

[102] Marzinelli E.M., Campbell A.H., Zozaya Valdés E., Vergés A., Nielsen S., Wernberg T., de Bettignies T., Bennett S., Caporaso J.G., Thomas T. (2015). Continental-scale variation in seaweed host-associated bacterial communities is a function of host condition, not geography. Environ. Microbiol..

[103] Wood G., Steinberg P.D., Campbell A.H., Vergés A., Coleman M.A., Marzinelli E.M. (2022). Host genetics, phenotype and geography structure the microbiome of a foundational seaweed. Mol. Ecol..

